# Exosomal lncRNA MIAT enriched in M2 macrophages contributes to electroacupuncture-driven peripheral nerve regeneration through targeting MiR-130a-3p/KLF7

**DOI:** 10.3389/fneur.2026.1765438

**Published:** 2026-05-01

**Authors:** Ming-yue Tian, Feng-xing Li, Hao Wang, Fang-fang Mou, Jing Zhu, Shui-jin Shao

**Affiliations:** School of Integrative Medicine, Shanghai University of Traditional Chinese Medicine, Shanghai, China

**Keywords:** electroacupuncture, exosomes, lncRNA MIAT, macrophage, peripheral nerve injury

## Abstract

**Aims:**

Electroacupuncture (EA) promotes the regeneration and repair of peripheral nerve injury (PNI) in clinical, and its underlying neuroimmune mechanisms have still not been fully clarified. Our study investigates the role of exosomal lncRNA MIAT in EA-mediated repair of PNI.

**Methods:**

The study observed EA’s effect on PNI in a rat model established by sciatic nerve injuries. The sciatic nerve function was observed via sciatic nerve function index. Serum exosomal and sciatic nerve lncRNA MIAT levels were quantified, macrophage polarization was detected via immunofluorescence. Interventions included local lentiviral lncRNA MIAT overexpression (LV-MIAT), adeno-associated virus-mediated lncRNA MIAT knockdown (AAV-siMIAT), and GW4869 (exosome inhibitor) injection. Neuronal cells were transfected with miR-130a-3p mimics/inhibitors or LV-MIAT, and lncRNA MIAT/miR-130a-3p/KLF7 interactions were confirmed via dual-luciferase assays.

**Results:**

EA intervention promotes peripheral nerve functional recovery in PNI rats, enhances axonal density, increases M2 macrophage number, and exosomal lncRNA MIAT level. M2-Exo administration in neuron promoted nerve cells viability and neurite length, while siMIAT/GW4869 partially reversed these effects. More, lncRNA MIAT acted as a miR-130a-3p sponge, relieving its repression of KLF mRNA and protein levels. Overexpressing lncRNA MIAT or silencing miR-130a-3p activated KLF7 expression, enhancing neuronal survival and axon regeneration.

**Conclusion:**

EA intervention improved PNI, restored neuronal function, increased PNI area M2 macrophage polarization and regulated lnc MIAT/miR-130a-3p/KLF7 axis expression.

## Introduction

Peripheral nerve injury (PNI) refers to damage to the nerve trunks and branches of the peripheral nervous system. In severe cases, PNI may result in permanent disability, imposing a significant economic burden on both patients and society (1, 2). However, nerve regeneration and function reconstruction remain clinical obstacles (3). Persistent challenges motivate investigation of the underlying molecular mechanisms to identify more effective interventions to nerve repair. Consequently, it is essential to search for novel key targets that address these unmet clinical needs.

Electroacupuncture (EA) is a treatment that combines traditional acupuncture with modern electrical stimulation, and it is an effective means to tackle PNI (4). EA can improve muscle strength of areas innervated by injured peripheral nerves, relieve the numbness and painful symptoms of PNI, and restore the motor functioning (5). The previous research also proved that EA promotes neuromotor function, muscle atrophy, and axonal regeneration after PNI (6–8). At present, it is widely accepted that EA can facilitate nerve regeneration by enhancing the function and proliferation of Schwann cells (SCs), inhibiting apoptotic activities in the injured neurons, regulating local blood flow, and reducing local inflammation (9, 10). However, the reproducibility of EA effects across studies is frequently undermined by variations in technical parameters. In the context of PNI treatment, researchers report differing selections of acupoints, stimulation frequencies, and current intensities. Despite this variability, a consistent observation is the predominant use of the ST36 (Zusanli) acupoint (5). More importantly, studies repeatedly demonstrate that EA at frequencies ranging from low (1 Hz) to high (100 Hz) promotes functional recovery, with higher frequencies proving to be more effective (11). These findings underscore the necessity of identifying the unifying biological mechanisms that transcend these technical differences.

The neuroscience community generally acknowledged that timely and effective regeneration of axons is essential for functional recovery after PNI, and two kinds of non-neuronal cells, SCs and macrophages, are contribute to remyelination, particularly macrophages (12). In recent years, growing attention has been paid to the function of macrophages in recovery after PNI (13). The repairing function of macrophages largely depends on its polarization properties. To respond signals from injured nerves, macrophages polarize to different functional properties, M1-polarized macrophages exhibit pro-inflammatory properties, but M2-polarized macrophages contribute to anti-inflammation and help promote PNI repair (14). Studies have shown that EA can inhibit macrophage polarization toward the M1 phenotype, reducing neural injury in mouse stoke models (15). EA also promotes polarization toward the M2 macrophage phenotype, thereby attenuating inflammation in multiple inflammatory conditions (16, 17). However, there is insufficient evidence on whether EA promotes PNI repair by regulating macrophage polarization, and the underlying molecular mechanism for M2 - polarized macrophages to help nerve regeneration was obscure.

Exosome is a type of membrane-enclosed nanoparticles, with a diameter of 30-150 nm, which containing multiple types of bioactive molecules, such as proteins, lipids, and nucleic acids (e.g., mRNAs, miRNAs, and lncRNAs) (18). As the mediators of intercellular communication, exosomes are widely present in various cells, including macrophages (19). Recently, the functions of exosomes secreted from macrophages in diverse diseases, such as autoimmune diseases and neurological diseases, have been extensively reported in the literature, especially those functions related to tissue repair, immunoregulation, and other physiological states (20, 21). Evidence suggests that exosomes serve as a key mediator for EA to mitigates neuronal degenerative damage (22). However, how exactly EA utilizes exosomal signaling to promote nerve repair remains unclear. Studies indicate that exosomes derived from M2 macrophages can expedite the repair of spinal and brain injury (23, 24). Many researchers assume that non-coding RNAs (ncRNAs) contents in the exosomes released by M2 macrophages are playing a role in macrophage-derived substances (25–27). lncRNAs that enriched in M2 macrophage-derived exosomes could be transferred to recipient cells and acted as an endogenous sponge to microRNA resulting in regulating the target gene expression (28).

MIAT (myocardial infarction-associated transcript), a long noncoding RNA (lncRNA), which is highly expressed in nervous system, and also participates in the damage repair of central nervous system (CNS) studies have shown (29). MIAT exists in the exosome (30). It has been reported that the expression level of MIAT significantly decreases after sciatic nerve transection (31). Transcriptome sequencing has shown that lncRNA MIAT is significantly downregulated in injured neural tissue, suggesting it may be a potential therapeutic target for promoting repair after PNI (32). Moreover, MIAT takes part in the regulation of inflammation in diverse diseases and might be involved in macrophages polarization (33). Previous study showed that MIAT was highly expressed in macrophages. Therefore, we propose that M2 macrophages promote PNI repair through exosome-mediated MIAT transport.

Given the lack of direct evidence, a preclinical study is warranted. The present study was designed to explore whether and how EA modulates macrophage polarization and exosomal communication to facilitate nerve repair. From investigating the mechanisms of EA treatment for PNI, this study demonstrated that EA intervention significantly upregulated the level of lncRNA MIAT and M2-polarized macrophages in sciatic nerve, and we proved that exosome-derived lncRNA MIAT was identified as a key mediator of EA-promoted repair after PNI through its role in polarizing local macrophages toward the M2 phenotype. Furthermore, biopredictive software (Targetscan/Starbase/miRDB) suggests lncRNA MIAT has multiple potential binding sites in miR-130a-3p (34). We delved into the synergetic mechanisms of MIAT, and found that lncRNA MIAT, enriched in exosomes from M2 macrophages, promotes neuronal axonal regeneration by targeting miR-130a-3p/KLF7.

## Materials and methods

### PNI rat model

All the experiments involving animals were conducted in full compliance with the *Guiding Opinions on Treating Laboratory Animals Kindly* (2006, 398) promulgated by the Ministry of Science and Technology of China. The Research Animal Ethics Committee of Shanghai University of Chinese Medicine reviewed and authorized animal research. The ethical approval number was PZSHUTCM2211010001. Male Wistar rats (SPF grade, weighing: 200 ± 20 g) were purchased from The Shanghai Slake Laboratory Animal Co., Ltd. (Shanghai, China), and raised by the Animal Experiment Center. The rats were maintained under a control condition (12-h dark/light cycle, temperature 22–25 °C, relative humidity 30–70%) with unrestricted access to food and water. Each rat was anesthetized with 2% pentobarbital sodium (at a dose of 40 mg/kg, intraperitoneal injection), then right sciatic nerve was transected from the middle thigh and immediately sutured. In the sham group, we only incised the skin and muscles of the rats to expose the sciatic nerve.

### EA intervention

**Figure 1:**
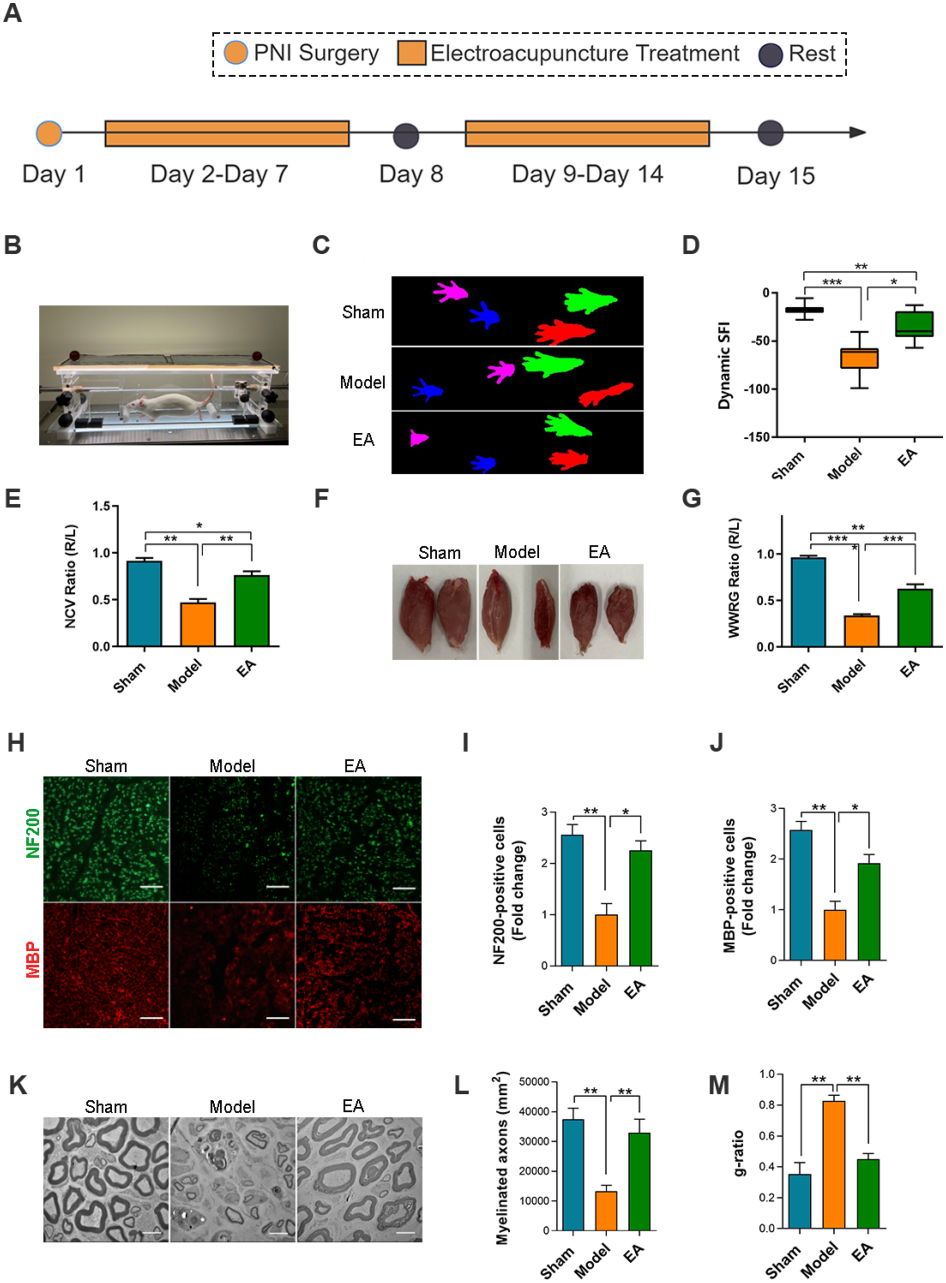
Electroacupuncture enhances peripheral nerve regeneration in rats. **(A)** Schematic of experimental workflow. **(B)** Behavioral experimental process of gait analysis. **(C)** Catwalk gait analysis system captured rat’s footprints. Red represents the affected foot. **(C–G)** Sciatic nerve functional recovery in rats: comparative analysis of sciatic function index, nerve conduction velocity recovery rate, and wet weight ratio of gastrocnemius in each group (*n* = 10). **(H)** Immunofluorescence staining of sciatic nerve: green (NF200, neuronal marker), red (MBP, myelin basic protein); scale bar = 100 μm. **(I,J)** Relative number of NF200^+^ cells **(I)** and MBP^+^ cells **(J)** were quantitatively analyzed using ImageJ software. Data expressed as relative fold change vs. sham group (*n* = 6). **(K)** Ultrastructural assessment by transmission electron microscopy (TEM); scale bar = 5 μm. **(L,M)** Morphometric analysis of myelinated axons: **(L)** myelinated axon count; **(M)** *g*-ratio [calculated as axon inner diameter ÷ total fiber diameter (axon + myelin)] (*n* = 3). Lower *g*-ratio indicates thicker myelin sheath. (Data are presented as arithmetic mean ± SEM. ^*^p < 0.05, ^**^p < 0.01, and ^***^p < 0.001).

The intervention was initiated 1 day after the modeling process. Rats in EA group accepted electroacupuncture stimulation once a day for 2 weeks (with 1 day off every 6 days), and each treatment would last for 20 min (Figure 1A). EA intervention was administered at GB30 (Huantiao) and ST36 (Zusanli) acupoints using the G6805A electroacupuncture apparatus (manufactured by Changzhou Yingdi Electronic Medical Equipment Co., Ltd.) with a frequency of 5 Hz discontinuous wave and 2 mA current intensity. Acupoint locations in rats were identified using the standardized atlas *Nomenclature and Location of Acupoints for Laboratory Animals—Part 2: Rat* (35). GB30 (Huantiao) is at the posterior–superior edge of the hip joint, and ST36 (Zusanli) lies on the posterolateral aspect of the knee joint, about 3 mm below the fibular head. In the model group and the sham group, rats were simply seized and restrained for 20 min each day.

### The sciatic nerve function assessment

The sciatic nerve function index (SFI) was analyzed by DigiGait imaging system (Mouse Specifics, Inc., Boston, United States). Rats were placed on a running platform and ran at a uniform speed of 10 mm/s for 10 s. When the rat’s paw makes contact with the surface of the platform, the footprint image is captured and recorded by a high-speed camera (Figure 1B). Using DigiGait imaging system to calculate parameters of the footprint image to get the SFI. The closer the SFI value is to 0, the better the function recovery.

The sciatic nerve conduction velocity was tested using RM6240 multi-channel biological signal acquisition system (Chengdu, China). The ratio of the velocity on the affected side to that on the healthy side is defined as the nerve conduction velocity recovery rate (NCV). The closer the NCV rate is to 1, the better the function recovery.

After SFI assay, all rats were sacrificed by isoflurane inhalation and 2% pentobarbital sodium (at a dose of 150 mg/kg, intraperitoneal injection), then cervical spinal dislocation after deep anesthesia, based on *Guidelines for euthanasia of laboratory animals*.

### Macrophages culture and induction

RMa-bm (rat macrophage line; HTX2703 ATCC) was maintained in RPMI-1640 medium (GIBCO 318000222) containing 10% FBS (Invitrogen C0235) and 1% penicillin–streptomycin under standard culture conditions (37 °C, 5% CO_2_ atmosphere). Macrophage subtypes were induced, when cell density reached approximately 50%. M1 phenotype was induced by 0.5 μg/mL lipopolysaccharide (LPS, Beyotime S1735), and M2 phenotype was established by administration of 20 ng/mL interleukin-4 (IL-4, Sigma I3650).

### NG108-15 cells culture and treatment

NG108-15 cell line (HB-12317; ATCC) was cultured in DMEM containing 10% FBS. The culture medium was further enriched with 0.1 mM hypoxanthine (Sigma H9377), 400 nM aminopterin (Sigma A1784), and 16 μM thymidine (Sigma T1895). The cells were seeded in 24-well or 96-well plates at an initial density of approximately 30%. Exosomes derived from three types of macrophage subtypes (M0/M1/M2-EXO) were supplemented into culture systems at 10 μg/mL. This intervention was initiated 24 h post-seeding when cells attained ~50% confluence in multi-well plates (24/96 wells) initially plated at 30% density, followed by 48-h incubation.

NG108-15 cell cultures underwent oxygen–glucose deprivation (OGD) intervention to establish a hypoxic–ischemic injury model. When cell confluence reaches 60–70%, the culture medium was replaced with glucose-free FBS-supplemented medium, followed by a 6-h incubation in a hypoxic chamber maintained at 37 °C. Following this incubation, the existing medium was aspirated prior to cell resuspension in fresh standard medium, followed by a subsequent 3-h culture phase under controlled conditions (37 °C, 5% CO2).

### Isolation and identification of exosomes

Serum-derived and macrophage-derived exosomes were isolated using differential ultracentrifugation. For the isolation of serum exosomes, blood was collected from the rat abdominal aorta and allowed to clot at room temperature for 4 h. The serum was then separated by centrifugation at 1,500 × g for 30 min at 4 °C. The supernatant underwent sequential centrifugation at 10,000 × g for 30 min and 14,000 × g for 30 min, both at 4 °C, to eliminate cells, debris, and large vesicles. The final supernatant was subjected to ultracentrifugation at 110,000 × g for 120 min at 4 °C. The supernatant underwent sequential centrifugation at 10,000 × g for 30 min and 14,000 × g for 30 min, both at 4 °C, to eliminate cells, debris, and large vesicles. The final supernatant was subjected to ultracentrifugation at 110,000 × g for 120 min at 4 °C using an ultracentrifuge (Hitachi; Japan). The exosome pellet was washed with cold 0.1 M PBS (pH 7.4) and centrifuged again at 110,000 × g for 60 min. Exosomes were resuspended in PBS, and their protein concentration was measured using the Enhanced BCA Protein Assay Kit (P0010, Beyotime). For macrophage-derived exosomes, exosomes were isolated from the culture supernatant of unstimulated (M0), M1-polarized, and M2-polarized macrophages, designated as M0-exo, M1-exo, and M2-exo, respectively. The cells were cultured in exosome-depleted medium (Opti-MEM^™^, Gibco). The conditioned medium was harvested and processed using the same ultracentrifugation protocol as described for serum exosomes. Exosome size was analyzed by nanoparticle tracking analysis (NTA) using the NanoSight LM10 system, and morphology was examined via Transmission Electron Microscopy (TEM; CM-10, Philips, The Netherlands). Exosome markers CD9 and CD63 were identified by Western blotting (Abcam, United Kingdom).

### MIAT transfection

Following the manufacturer’s instructions, Lipofectamine 2000 (Invitrogen, 11668019, Carlsbad, California, United States) was used to transfect M2 macrophages with small interfering MIAT (siMIAT) and a CD63-GFP plasmid. The siMIAT sequences are detailed in Table 1. NG108-15 cells were transfected with a miR-130a-3p mimic and inhibitor (RioBio, Guangzhou, China) using Lipofectamine 2000. Furthermore, NG108-15 cells were transduced with a lentivirus overexpressing MIAT (BrainVTA, Wuhan, China), using an innovative infection enhancer (Enhancer A + B, Wuhan, BrainVTA).

**Table 1:** Sequence.

siMIAT sequence siRNA	Sequence
siMIAT-1	GGGAUUGGGAAAGGUCAUATTUAUGACCUUUCCCAAUCCCTT
siMIAT-2	GCUCCUUAUGCUGUUUAUATTUAUAAACAGCAUAAGGAGCTT
siMIAT-3	GUCCCCUAAUUCCACUGAATTUUCAGUGGAAUUAGGGGACT

To manipulate MIAT expression *in vivo*, we used lentiviral vectors for overexpression (LV-MIAT, BrainVTA, Wuhan, China) and adeno-associated viral vectors (AAV-siMIAT, GeneChem, Shanghai, China) for knockdown, each at a titer of 1 × 10^8^ transducing units (TU)/mL. For overexpression, 50 μL of undiluted LV-MIAT suspension was injected into the sciatic never concurrently with the modeling procedure. For knockdown, AAV-siMIAT was mixed with an equal volume (25 μL) of Matrigel (BD Biosciences, United States) and administered as a 50 μL total volume 2 weeks prior to modeling, the schematic timeline of the experimental design shown as Figures 1A,5A,6A. Control animals received equivalent injections of the corresponding empty-vector viruses.

### GW4869 treatment

To inhibit exosome secretion *in vivo*, we used the exosome biogenesis inhibitor GW4869 (Sigma-Aldrich, United States). From separate dilutions of a GW4869/DMSO stock (8 mg/mL) and of pure DMSO in saline, we prepared two working solutions, one containing 0.3 mg/mL GW4869 (in 3.75% DMSO) and the other containing 3.75% DMSO as the control. Rats in the treatment group received intraperitoneal injections of this solution at 1 mg/kg body weight every other day for 2 weeks, concurrent with the EA treatment. Control animals received intraperitoneal injections of 3.75% DMSO.

### Exosomal MIAT uptake analysis

Exosomes were collected from M2 macrophages transfected with a CD63-GFP plasmid and co-cultured with NG108-15 cells for 48 h. Fluorescence *in situ* hybridization combined with immunofluorescence staining was then used to detect the transfer of exosomal MIAT from macrophages to NG108-15 cells.

### Quantitative real-time PCR (qPCR)

Total RNA from cells and sciatic nerve tissues were extracted using NucleoZol (740404.6, MACHEREY-NAGEL). For reverse transcription, the miRcute Plus miRNA First-Strand cDNA Synthesis Kit and FastKing gDNA Dispelling RT Super-Mix Kit (KR211-02 and KR118-02, Tiangen, Beijing, China) were employed to convert miRNAs and total RNA into cDNA. miRNA and mRNA quantification were performed separately using the miRcute Plus miRNA qPCR Kit (SYBR Green) (FP411, Tiangen, Beijing, China) and Hieff® qPCR SYBR Green Master Mix (High Rox Plus) (11203ES08, Yeasen, Shanghai, China), respectively. Primer sequences are listed in Table 2. Data analysis was conducted using the 2^–ΔΔCT^ method.

**Table 2:** Primers.

Gene	Forward	Reverse
CD86	CAACTAATGAGTATGGCGACA	GGGAATGGAAGAGATAGGCT
TNF-α	CCAATCTGTGTCCTTCTAACT	TGTGTTTCTGAGCATCGT
IL-1β	GTTCTTTGAGGCTGACAGACC	GATGCTGCTGTGAGATTTGAA
LNC MIAT	GCTGAAGTGCCAGCAGTAGC	
GAPDH	ATGACTCTACCCACGGCAAG	GGAAGATGGTGATGGGTTTC
CD11b	CTGGGAGATGTGAATGGAG	ACTGATGCTGGCTACTGATG
CD86	CAACTAATGAGTATGGCGACA	GGGAATGGAAGAGATAGGCT
iNOS	GTGAGGAGCAGGTTGAGGATT	GAAAAGACCGCACCGAAGAT
Arg1	CGGGAAGGTAATCATAAGCCA	GTTCTGTTCGGTTTGCTGTGA
MRC1	CCTTCTGTGCCTATCTCTCCA	TATTTCTCTGCTTCGTGCCAT
CD163	GCAGCTCTCACTGGGACATAG	GAAAGGGCAACTCCACACTTA
TNF-α	CCAATCTGTGTCCTTCTAACT	GTGTTTCTGAGCATCGT

### Immunofluorescence staining (IF)

Cultured macrophages were collected and spread onto slides. The cells were fixed with 4% paraformaldehyde for 20 min, followed by permeabilization with 0.5% Triton X-100 for 15 min at room temperature. Primary antibodies were applied and incubated overnight at 4 °C. After incubation, cells were blocked with a secondary antibody and mounted using an anti-fade mounting medium containing 4′,6-diamidino-2-phenylindole (DAPI) (P0131, Beyotime, Shanghai). The primary antibodies used are listed in Table 3. Fluorescent images were captured using a fluorescence microscope, and ImageJ software was utilized for statistical analysis.

**Table 3:** Antibodies.

Antibody	Company
Anti-NF200	Sigma-Aldrich
Anti MBP	Acam
CD206/MRC1 (E6T5J)	CST
CD9	Abcam
CD63	Abcam
β3-tubulin	Abcam
Anti-F4/80	Abcam
Anti-iNOS	Abcam
Anti-GAPDH	Proteintech

### Fluorescence *in situ* hybridization (FISH)

The specific fluorescent probes for MIAT, with the sequence ATCTCCCCTTCAGAGTCCCC, were obtained from Guangzhou, China. The fluorescence *in situ* hybridization kit for RNA (R0306S, Beyotime, Shanghai) was used to detect MIAT in NG108-15 cells. By co-labeling with VD63-GFP, exosomal MIAT delivery to NG108-15 cells was demonstrated. The specific operational steps followed the kit instructions.

### The dual-luciferase reporter assay

The binding sites of miR-130a-3p on lncRNA MIAT and KLF7 were identified. Portions of lncRNA MIAT and KLF7, containing either the wild-type or mutant miR-130a-3p binding sequences, were cloned into a pGL3 promoter vector (Genechem, Shanghai, China) to construct MIAT-WT, MIAT-MUT, KLF7-WT, and KLF7-MUT plasmids. NG108-15 cells were seeded into 24-well plates and transfected with 5 nM Renilla luciferase vector, 50 nM miR-130a-3p mimics, and a negative control using Lipofectamine 2000 (Invitrogen, 11668019, United States). After 48 h of transfection, the cells were lysed, and luciferase activity was measured using the Dual-Luciferase Reporter Assay System (Beyotime, RG027, Shanghai, China) to assess the regulatory effects.

### Western blot

Total protein from sciatic nerves, exosomes, and cells was extracted using a total protein extraction kit (BC3710, Solarbio, Beijing). Quantitative protein analysis was conducted utilizing the enhanced BCA assay Kit (P0010, Beyotime Biotechnology, Shanghai). Protein lysates were combined with 5× denaturing loading buffer and separated using a 12.5% SDS-PAGE system (NCM Biotech, Suzhou). The separated proteins were then transferred to polyvinylidene fluoride (PVDF) membranes (FFP28, Millipore, United States). The membranes were incubated overnight at 4 °C with the primary antibody, followed by incubation with the appropriate secondary antibody. Detailed information on the antibodies used is provided in Table 3. Protein expression levels were detected using enhanced chemiluminescence (P10100, NCM Biotech, Suzhou). The gray values of the protein bands were quantified using ImageJ software for statistical analysis.

### Statistical analysis

Statistical analyses were conducted with GraphPad Prism 8.0. Blinding was applied to all quantitative histological assessments. Blinding was applied to all quantitative histological assessments, animal codes and group assignments were disclosed only after completing all data analyses. Quantitative data are presented as mean values with standard error of the mean (SEM). For intergroup comparisons, one-way ANOVA was performed with Tukey’s *post-hoc* test to assess specific pairwise differences. A significance threshold of *p* < 0.05 was applied for all statistical determinations.

## Results

### EA can promote nerve repair after PNI

To assess the effectiveness of EA in the recovery of motor function in rats, we conducted gait analysis to evaluate the sciatic nerve function index (SFI) and performed electrophysiological experiments to measure the recovery rate of nerve conduction velocity (NCV). Results from the DigiGait imaging system revealed that the Sham group had clearly visible footprints with fully spread toes. In contrast, the Model and EA groups showed noticeable toe contraction in their footprints. However, the EA group demonstrated a greater degree of toe spreading compared to the Model group (Figure 1C). Additionally, the SFI significantly increased in the EA group, suggesting that EA can substantially enhance the sciatic nerve function index following PNI (Figure 1D). The nerve conduction velocity in the EA group was significantly higher than that in the Model group (Figure 1E). To assess denervation-induced muscle atrophy, we compared the wet weight ratios of freshly harvested gastrocnemius muscles (WWRG) in rats. Both the Model and EA groups exhibited varying degrees of atrophy in the affected limb, except for the Sham group. Notably, the EA group demonstrated significant improvement, suggesting that EA effectively alleviates muscle atrophy following PNI (Figures 1F,G). An Immunofluorescence analysis revealed that EA intervention can effectively increase the number of neuronal axons and myelin in the cross-sections of the injured sciatic nerve (Figures 1H–J). Transmission electron microscopy revealed that the number of axons and the myelin sheath thickness in the injured sciatic nerve of the EA group were significantly increased compared to the Model group (Figures 1K–M). All together, these results suggest that EA intervention can significantly promote nerve repair following PNI.

### EA drives macrophages to M2 phenotype after PNI

**Figure 2:**
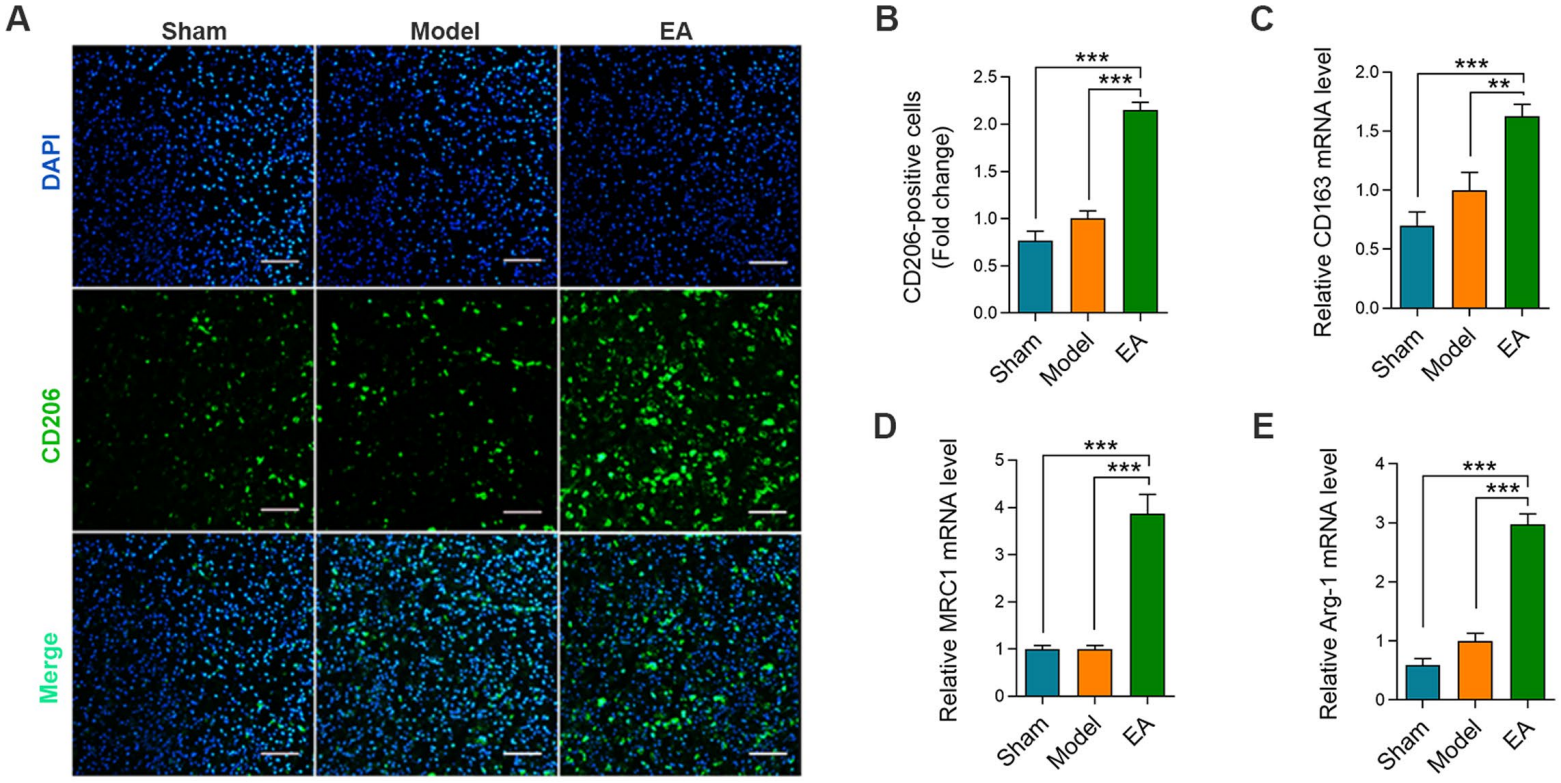
Assessment of M2 macrophage polarization in sciatic nerve tissue. **(A)** Immunofluorescence staining of CD206 (M2 macrophage marker); scale bar = 100 μm. **(B)** Quantitative analysis of CD206^+^ cell relative number by ImageJ, expressed as relative fold change vs. sham group (*n* = 6). **(C–E)** mRNA expression levels of M2 markers CD163 **(C)**, Arg-1 **(D)**, and MRC1 **(E)** by RT-qPCR (*n* = 5). Data normalized to GAPDH. (Data are presented as arithmetic mean ± SEM. ^**^p < 0.01 and ^***^p < 0.001).

To investigate the polarization trend of macrophages in nerve tissue following EA intervention, we conducted immunofluorescence staining on sections of the injured sciatic nerve. The results showed that after electroacupuncture intervention, the number of CD206^+^ cells in the cross-sections of the injured sciatic nerve was significantly increased (Figures 2A,B). Furthermore, qPCR analysis corroborated these results, indicating that the mRNA expression levels of M2 phenotype macrophages in the EA group were markedly higher than those in the Model group (Figures 2C–E). These results suggest that EA facilitates the polarization of macrophages toward the M2 phenotype in the sciatic nerve.

### The induction of M1/M2 macrophages and identification of exosomes

**Figure 3:**
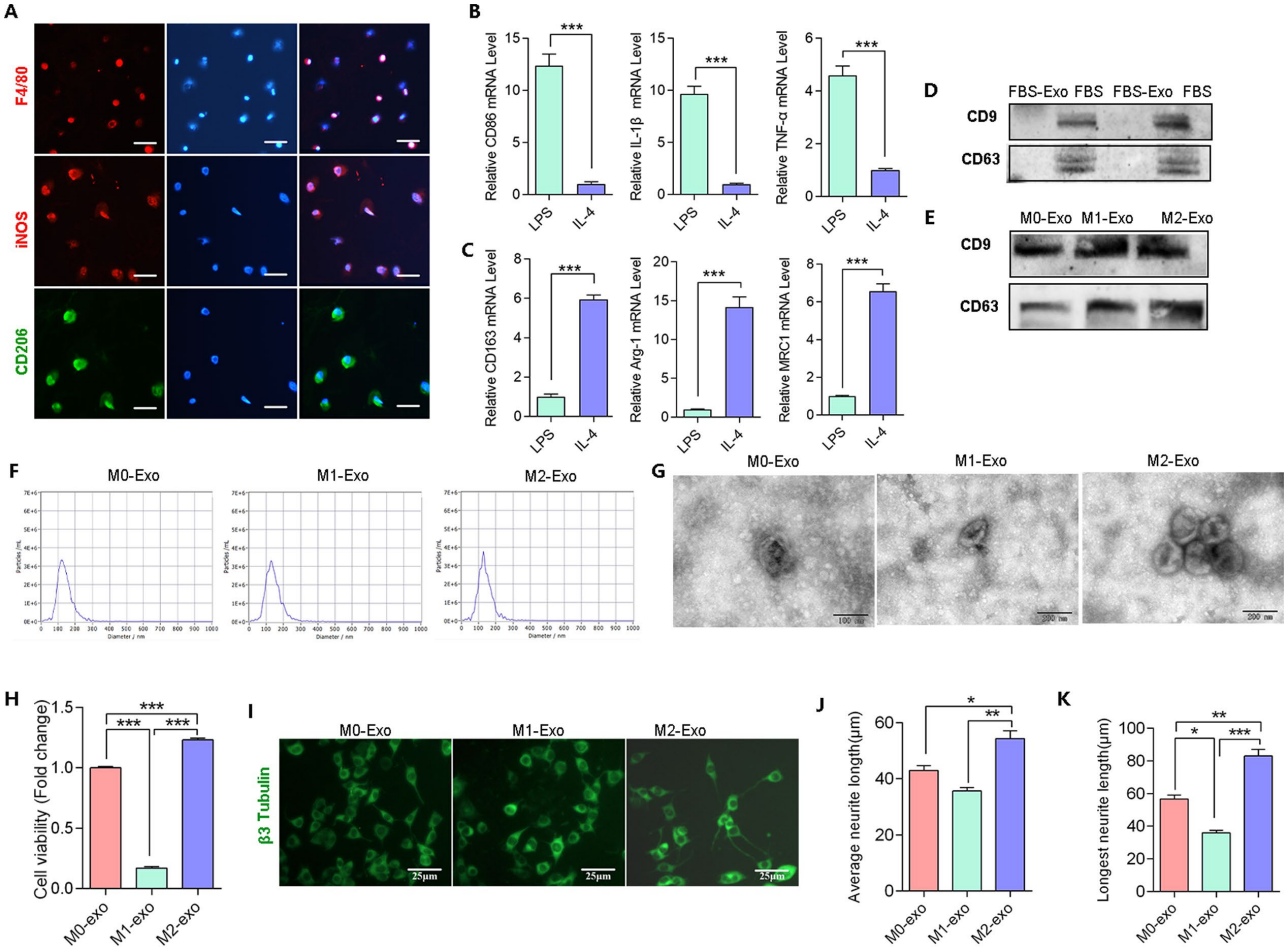
Characterization of exosomes derived from different macrophage phenotype. **(A)** Immunofluorescence staining of M0/M1/M2 macrophage markers (red: F4/80, iNOS; green: CD206); scale bar = 25 μm. **(B)** mRNA expression of M1 markers (CD86, IL-1β, TNF-α) and M2 markers (CD163, Arg-1, MRC1) in PNI nerve tissue by RT-qPCR. Data normalized to GAPDH (*n* = 5). **(D,E)** Western blot analysis of exosomal markers CD9 and CD63: **(D)** exosome-depleted FBS; **(E)** different macrophage-derived exosome. **(F)** Nanoparticle tracking analysis (NTA) showing exosome size distribution, the particle size of exosome extracted from macrophages is about 50–150 nm, total median particle number concentration was 3 × 10^5^ particles/mL. **(G)** Transmission electron microscopy (TEM) revealing exosome morphology. The exosome preparations were cup-shaped and measured approximately 100 nm in diameter: scale bar = 200 nm. **(H)** Effect of exosomes derived from distinct macrophage phenotypes on NG108-15 cell viability assessed by CCK-8 analysis. **(I)** Immunofluorescence staining of neuronal cytoskeleton (βIII-tubulin); scale bar = 25 μm. **(J,K)** Quantification of neurite outgrowth in NG108-15 cells: mean **(J)** and maximal **(K)** lengths (*n* = 6, data measured by ImageJ). (Data are presented as arithmetic mean ± SEM. ^*^p < 0.05, ^**^p < 0.01, and ^***^p < 0.001).

To explore the effects of exosomes from different macrophage phenotypes on nerve growth, we conducted *in vitro* experiments. Macrophages were stimulated with LPS to induce M1 and with IL-4 to induce M2 phenotypes. This was confirmed by immunofluorescence staining, which showed that unstimulated macrophages (M0) expressed the marker F4/80, while LPS-induced macrophages highly expressed the M1 marker iNOS, and IL-4-induced macrophages expressed the M2 marker CD206 (Figure 3A). RT-qPCR analysis revealed that LPS stimulation significantly upregulated M1 markers CD86, TNF-α, and IL-1β (Figure 3B), whereas M2 markers CD163, MRC1, and Arg1 were downregulated (Figure 3C). These results confirm the successful induction of M1 and M2 macrophages. With the next step, we collected exosomes from M0, M1 and M2 macrophage and characterized the isolated exosomes using Western blotting, transmission electron microscopy (TEM), and nanoparticle tracking analysis (NTA) (Figures 3D–G).

### M2-derived exosomes promote neural cells growth

To further investigate the impact of exosomes from different macrophage phenotypes on neural cells, we separately co-cultured NG108-15 cells with M0-exo, M1-exo and M2-exo for 48 h. The results of CCK8 assays showed that compared to M0-exo, M2-exo enhanced the viability of NG108-15 cells, while M1-exo dramatically inhibited the viability of NG108-15 cells (Figure 3H). Additionally, to observe neurite growth, we performed immunofluorescence staining on NG108-15 cells after 48 h of co-culture. As shown in Figure 3I. The average axon length (Figure 3J) and longest axon length (Figure 3K) of NG108-15 cells co-cultured with M2-exo were significantly greater than those in the other two groups. These results indicate that M2-exo can promote cell activity and axon growth of NG108-15 cells.

### M2-exo-lncRNA MIAT transfer to NG108-15 cells

**Figure 4:**
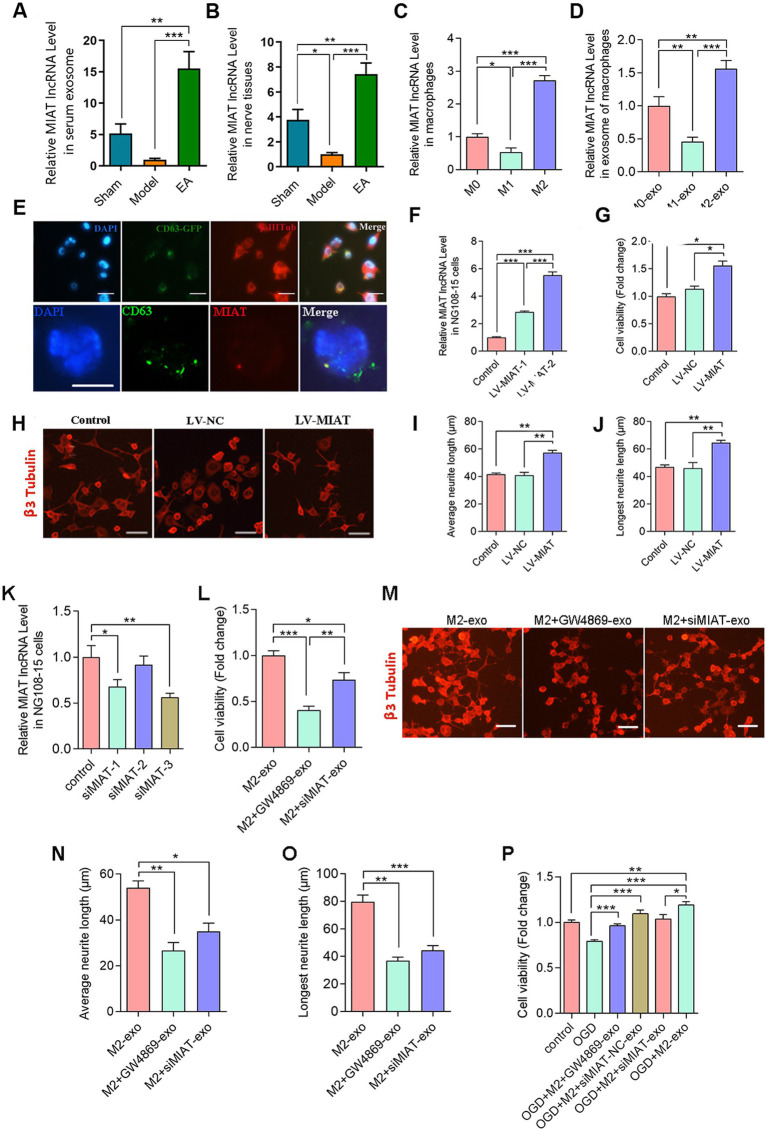
Exosomal lncRNA MIAT from M2 macrophages enhances NG108-15 cell viability and morphogenesis. RT-qPCR analysis of lncRNA MIAT expression in serum exosome **(A)**, sciatic nerves **(B)**, macrophages **(C)**, and exosome derived from macrophages **(D)**. Data normalized to U6 or GAPDH (*n* = 5). **(E)** Tracking of exosomal lncRNA MIAT transfer from M2 macrophages to NG108-15 cells via FISH-immunofluorescence co-localization analysis. **(F)** LV-MIAT-mediated MIAT overexpression efficiency analysis by RT-qPCR in NG108-15 cells. Data normalized to GAPDH. **(G)** NG108-15 cell viability assessment via CCK-8 assay under MIAT overexpression. **(H)** Neuronal cytoskeletal architecture visualized by βIII-tubulin immunofluorescence (Alexa Fluor 594, red). Scale bar = 25 μm. **(I,J)** Quantitative morphometrics: **(I)** mean neurite length; **(J)** maximum neurite extension. Analyzed by ImageJ. **(K)** RT-qPCR analysis of MIAT knockdown efficiency by siMIAT transfection (siMIAT-1/2/3) in NG108-15 cells. Data normalized to GAPDH (*n* = 5). **(L)** NG108-15 cell viability assessment via CCK-8 assay under MIAT silencing. **(M)** Neuronal cytoskeletal architecture visualized by βIII-tubulin immunofluorescence (Alexa Fluor 594, red). Scale bar = 25 μm. **(N,O)** Quantitative morphometrics: **(N)** mean neurite length; **(O)** maximum neurite extension. Analyzed by ImageJ. **(P)** CCK-8 assay detects the viability of OGD-treated NG108-15 cells. (Data are presented as arithmetic mean ± SEM. ^*^p < 0.05, ^**^p < 0.01, and ^***^p < 0.001).

To uncover the molecular mechanisms of EA-mediated exosome regulation after PNI, we centered our attention on the role of lncRNA MIAT in M2-exo. We detected a significant amount of MIAT expression both in the serum exosomes (Figure 4A) and the sciatic nerve (Figure 4B) of PNI rats after EA intervention. Notably, the expression level in the EA group was markedly higher than that in the model and sham groups. Furthermore, in the M2 macrophages, we also detected a large amount of lncRNA MIAT expression, and the expression level of MIAT in M2 was higher than that in M0 and M1 (Figure 4C). This trend was also further verified in the macrophage exosomes (Figure 4D), indicating that lncRNA MIAT exists in M2 and is expressed within M2-exo.

To demonstrate that MIAT is secreted by M2 macrophages and delivered to neural cells via exosome pathways, we first transfected pCT-CD63-GFP plasmid into M2 macrophages and then co-cultured the transfected M2-derived exosomes with NG108-15 cells. The results demonstrated by immunofluorescent staining showed that CD63-GFP was taken up by NG108-15 cells (Figure 4E, the first row). In order to colocalize CD63 with lncRNA MIAT in the previously co-cultured NG108-15 cells, we constructed a lncRNA MIAT fluorescent probe. The imaging combining fluorescence *in situ* hybridization (FISH) with anti-CD63 immunofluorescence revealed distinct co-localization patterns between CD63 and MIAT probe signals in cytoplasmic vesicles (Figure 4E, the second row). This observation provides direct evidence that M2-exo-lncRNA MIAT was internalized by NG108-15 cells.

### LV-MIAT transfection affected NG108-15 cells

To further validate the effect of lncRNA MIAT on NG108-15 cells, we established the NG108-15 cell line stably overexpressing MIAT by lentiviral transduction. The optimal transduction efficiency was observed at an MOI of 100, as determined by qPCR analysis 72 h post-transfection (Figure 4F). The CCK-8 assay demonstrated that MIAT overexpression led to a statistically significant increase in NG108-15 cell viability compared to negative control groups (Figure 4G). Immunofluorescence staining of β3-tubulin was performed to evaluate neuronal cytoskeletal morphology of NG108-15 cells, revealing that LV-MIAT transduction substantially enhanced neuritogenesis compared to control groups (Figures 4H–J). These findings support our hypothesis that overexpression of lncRNA MIAT promotes NG108-15 cell viability and neurite outgrowth.

### siMIAT transfection affected NG108-15 cells

M2 macrophages were transfected with siMIAT plasmids to further investigate the functional role of M2-Exo-lncRNA MIAT in NG108-15 cells. After comparing the knockdown efficiencies of multiple siMIAT plasmids, siMIAT-3 was selected for subsequent experiments based on its superior knockdown efficiency (Figure 4K). In parallel, M2 macrophages were treated with exosome inhibitor (GW4869). Following the 48-h co-culture of NG108-15 cells with M2-exo subjected to different treatments, CCK-8 assay revealed that both GW4869 and siMIAT-3 significantly suppressed NG108-15 cell viability (Figure 4L). This was also demonstrated in the immunofluorescence images (Figures 4M–O). To further validate the role of M2-exo-lncRNA MIAT, NG108-15 cells were subjected to oxygen–glucose deprivation (OGD) modeling. The results showed that OGD exposure significantly decreased neuronal cell viability, while M2-exo treatment alleviated OGD-induced cytotoxicity. Both GW4869 and siMIAT abolished the neuroprotective effects of M2-exo (Figure 4P).

### LV-MIAT administration in rats promote nerve regeneration

**Figure 5:**
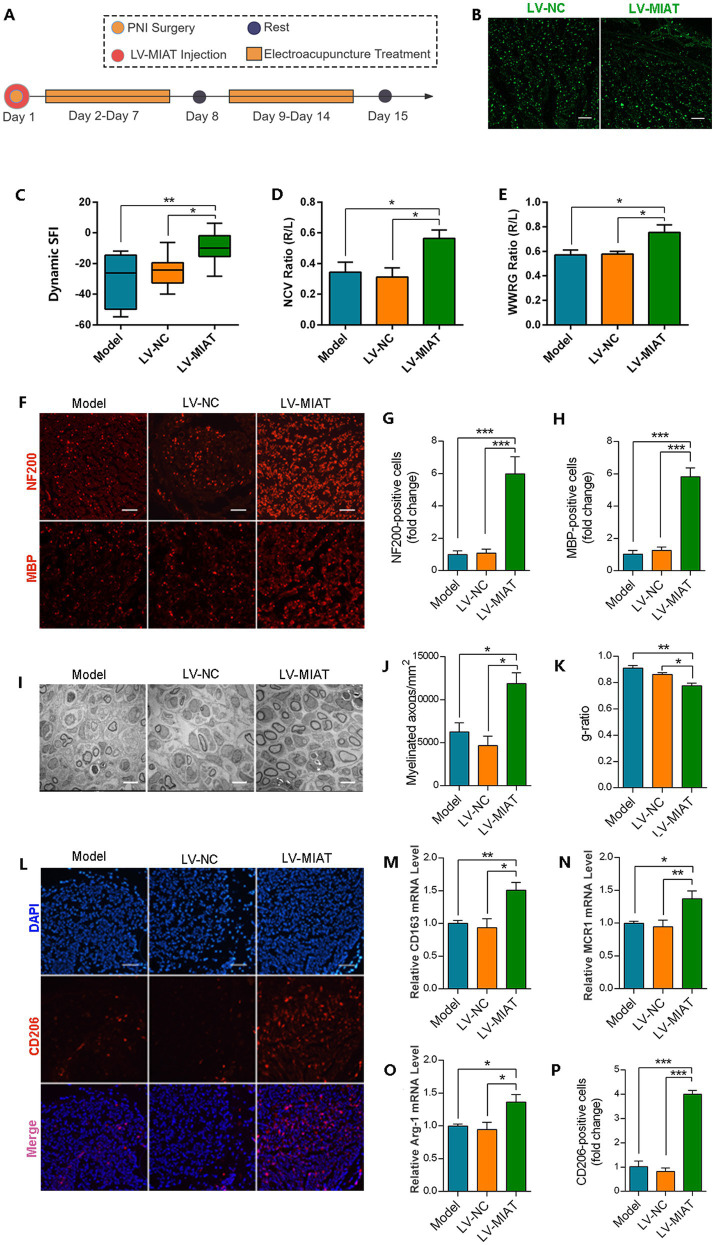
Regulatory role of lncRNA MIAT in post-PNI nerve regeneration and M2 macrophage polarization. **(A)** Schematic of experimental workflow. **(B)** Transduction efficiency of LV-MIAT lentivirus localized at the PNI site (scale bar = 100 μm). **(C–E)** Sciatic nerve functional recovery in rats: comparative analysis of sciatic function index, nerve conduction velocity recovery rate, and wet weight ratio of gastrocnemius in each group (*n* = 10). **(F)** Immunofluorescence staining of NF200 (neuronal marker) and MBP (myelin basic protein) in sciatic nerves; (scale bar = 100 μm). **(G,H)** Relative number of NF200^+^ cells **(G)** and MBP^+^ cells **(H)** were quantitatively analyzed by ImageJ software. Data expressed as relative fold change vs. sham group (*n* = 6). **(I)** Ultrastructural assessment by transmission electron microscopy (TEM) (scale bar = 5 μm). **(J,K)** Morphometric analysis of myelinated axons: **(J)** myelinated axon count; **(K)** *g*-ratio [calculated as axon inner diameter ÷ total fiber diameter (axon + myelin)] (*n* = 3). Lower *g*-ratio indicates thicker myelin sheath. **(L–P)** M2 macrophage polarization dynamics in PNI: **(L)** representative immunofluorescence images of CD206^+^ M2 macrophages at the PNI region (scale bar = 100 μm). **(M–P)** RT-qPCR analysis of M2 phenotypic markers (CD163, MRC1, Arg-1, CD206) in distal nerve segments, normalized to GAPDH (*n* = 5). (Data are presented as arithmetic mean ± SEM. ^*^p < 0.05, ^**^p < 0.01, and ^***^p < 0.001).

To verify the effect of lncRNA MIAT in PNI repair, rats were injected with LV-MIAT or LV-NC immediately followed modeling. This approach was chosen for its ability to effectively deliver genetic material to target macrophages and progenitor cells (36), ensuring that overexpression coincides with the crucial phase of macrophage polarization during the EA intervention period. The scores of SFI, NCV, and WWRG were evaluated 2 weeks after lentivirus injection (Figure 5A). Fluorescent images of the nerve segments showed the lentivirus successfully infected the target tissues (Figure 5B). Local overexpression of lncRNA MIAT significantly promoted the motor capacity of PNI rats, not only improving neurological function but also alleviating muscle atrophy (Figures 5C–E). Axonal morphology was evaluated through NF200 immunofluorescence staining, while myelin sheath integrity was assessed via MBP immunofluorescence staining (Figure 5F). The results demonstrated significantly increased numbers of NF200^+^ cells and MBP^+^ cells in the LV-MIAT1 group, indicating that local overexpression of lncRNA MIAT effectively enhances axonal myelination regeneration capacity following PNI (Figures 5G,H). Furthermore, the TEM evaluation demonstrated that LV-MIAT intervention induced pronounced ultrastructural modifications, with both the quantity and thickness of myelin sheaths in the LV-MIAT group exhibiting a significant increase (Figures 5I–K).

### LV-MIAT administration in rats promote M2 macrophage polarization

Results from *in vitro* experiments demonstrated an interaction between MIAT and M2 macrophages. To confirm that lncRNA MIAT overexpression promotes macrophage M2 polarization, we performed immunofluorescence staining of CD206 on nerve tissues from PNI rats (Figures 5L,P). qPCR analysis of macrophage marker gene expression in sciatic nerve tissues revealed a trend consistent with the previous observations (Figures 5M–O). Collectively, these data demonstrate that LV-MIAT local administration in rat peripheral nerves mimics EA’s therapeutic effects by promoting nerve regeneration and.

### Exo-lncRNA MIAT mediates EA-promoted PNI recovery

**Figure 6:**
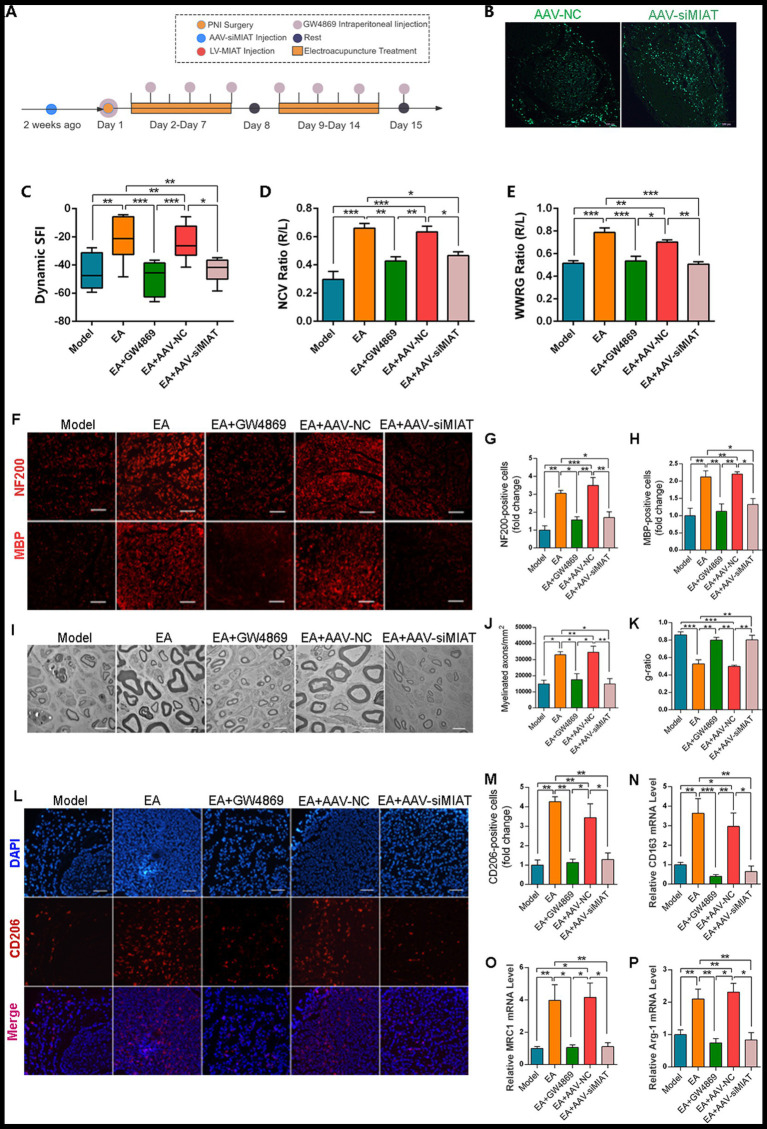
Knockdown of lncRNA MIAT impact nerve regeneration and M2 macrophage polarization. **(A)** Schematic of experimental workflow. **(B)** Transduction efficiency of siMIAT knockdown of MIAT at the PNI site, scale bar = 100 μm. **(C–E)** Sciatic nerve functional recovery in rats: comparative analysis of sciatic function index, nerve conduction velocity recovery rate, and wet weight ratio of gastrocnemius in each group (*n* = 10). **(F)** Immunofluorescence staining of NF200 (neuronal marker) and MBP (myelin basic protein) in sciatic nerves scale bar = 100 μm. **(G,H)** Relative number of NF200^+^ cells **(G)** and MBP^+^ cells **(H)** were quantitatively analyzed by ImageJ software. Data expressed as relative fold change vs. sham group (*n* = 6). **(I)** Ultrastructural assessment by transmission electron microscopy (TEM); scale bar = 5 μm. **(J,K)** Morphometric analysis of myelinated axons: **(J)** myelinated axon count; **(K)** *g*-ratio [calculated as axon inner diameter ÷ total fiber diameter (axon + myelin)] (*n* = 3). Lower *g*-ratio indicates thicker myelin sheath. **(L–P)** M2 macrophage polarization dynamics in PNI: **(L)** representative immunofluorescence images of CD206^+^ M2 macrophages at the PNI region (scale bar = 100 μm). **(M–P)** RT-qPCR analysis of M2 phenotypic markers (CD163, MRC1, Arg-1, CD206) in distal nerve segments, normalized to GAPDH (*n* = 5). (Data are presented as arithmetic mean ± SEM. ^*^p < 0.05, ^**^p < 0.01, and ^***^p < 0.001).

To investigate the role of exo-lncRNA MIAT in EA-facilitated PNI repair, we performed localized knockdown of lncRNA MIAT in rat sciatic nerves using adeno-associated virus (AAV) injection and exosome inhibitor treatment, respectively (Figure 6A). The transfection efficacy of AAV was confirmed by fluorescence microscopy (Figure 6B). Notably, both lncRNA MIAT knockdown and exosome inhibition significantly attenuated EA’s therapeutic effect on PNI motor capacity recovery, however, the NCV of Model group was lower than that of EA + AAV-siMIAT group and EA + GW4869 group (Figures 6C–E). Results of immunofluorescence staining showed that, compared to the EA group, both lncRNA MIAT knockdown and exosome inhibition obstructed axonal regeneration and myelin formation to a level that was not statistically different from the Model group (Figures 6F–H). In addition, the axons in both EA + AAV-siMIAT group and EA + GW4869 group had thinner myelin, observing under electron microscopy (Figures 6I–K). The previously obtained results that EA promoted macrophages polarizing toward M2 phenotype were significantly attenuated by either lncRNA MIAT knockdown or GW4869-mediated exosome inhibition, indicating the essential role of exo-lncRNA MIAT in mediating EA’s immunomodulatory effects during PNI repair (Figures 6L–P).

### lncRNA MIAT directly targets miR-130a-3p

**Figure 7:**
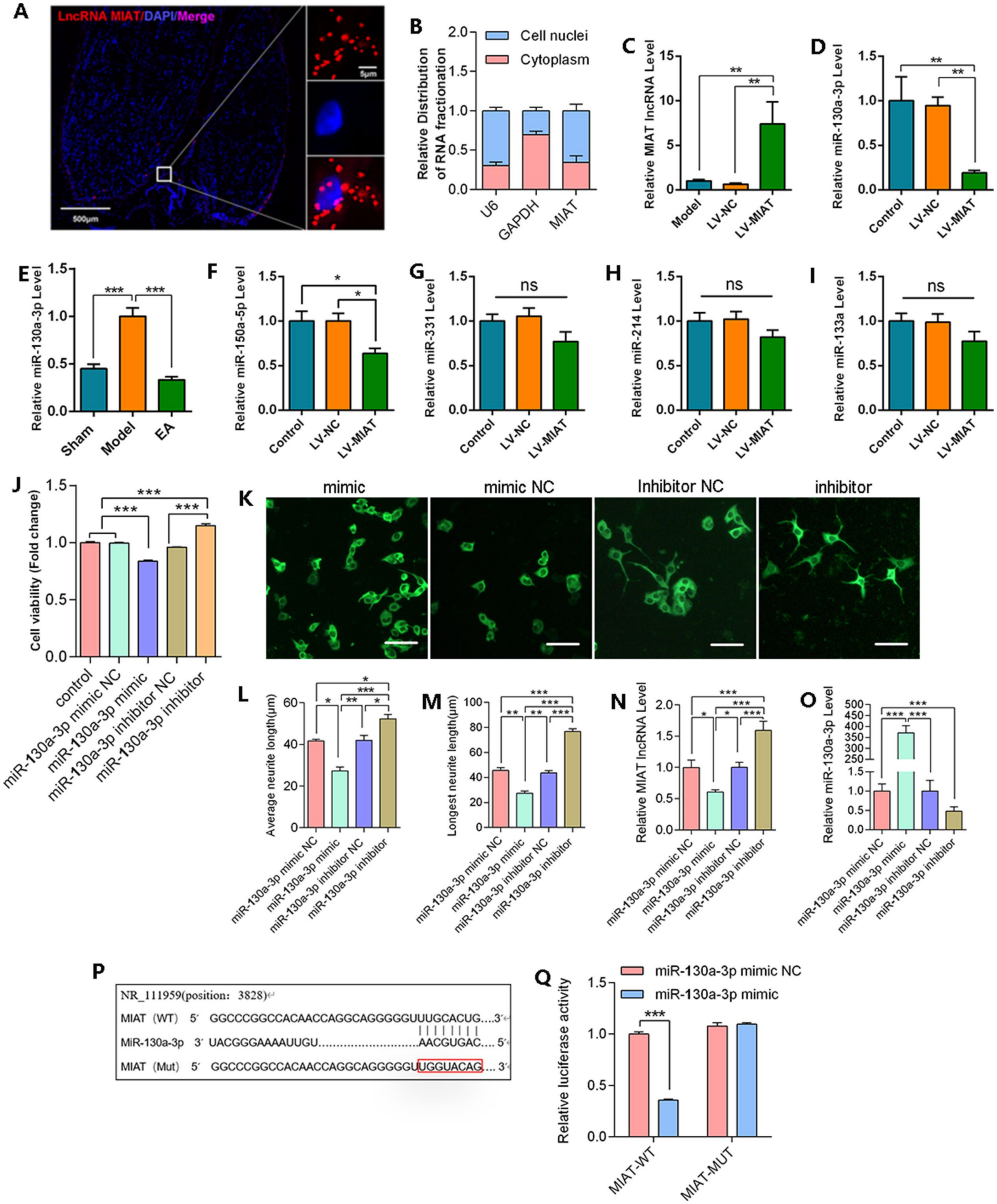
MiR-130a-3p modulates NG108-15 cell viability and neurite growth. **(A)** Observation of lncRNA MIAT subcellular distribution in local PNI tissue sites using FISH. **(B)** Detection of nuclear/cytoplasmic distribution of lncRNA MIAT by RT-qPCR. **(C)** RT-qPCR quantification of lncRNA MIAT overexpression efficiency **(D)** RT-qPCR analysis of miR-130a-3p expression levels in MIAT-overexpressing PNI tissues. **(E–I)** RT-qPCR analysis of candidate miRNAs (miR-130a-3p, miR-150a-5p, miR-331, miR-214, and miR-33a) expression in MIAT-overexpressing PNI tissues. Data normalized to U6 (*n* = 5). **(J)** CCK-8 assay evaluating the effect of miR-130a-3p transfection on NG108-15 cell viability. **(K)** Neuronal cytoskeletal architecture visualized by βIII-tubulin immunofluorescence (Alexa Fluor488, green). **(L,M)** Quantitative morphometrics: **(L)** mean neurite length; **(M)** maximum neurite extension. Analyzed by ImageJ. **(N)** RT-qPCR analysis of lncRNA MIAT expression on miR-130a-3p transfected NG108-15 cell. Data normalized to GAPDH (*n* = 5). **(O)** RT-qPCR analysis of miR-130a-3p expression levels. Data normalized to U6 (*n* = 5). **(P)** Bioinformatic prediction of interaction sites between lncRNA MIAT and miR-130a-3p. **(Q)** Dual-luciferase reporter assay validating the direct binding of lncRNA MIAT to miR-130a-3p (*n* = 5). (Data are presented as arithmetic mean ± SEM. ^*^p < 0.05, ^**^p < 0.01, and ^***^p < 0.001).

To further address the functional mechanism, the target transcribed genes of lncRNA MIAT wer investigated. Fluorescence *in situ* hybridization (FISH) technique is used to identify the lncRNA MIAT in injured peripheral nerve, and the result demonstrated that it was distributed in both the cytoplasm and nucleus (Figure 7A). The nucleocytoplasmic RNA fractionation validated the result (Figure 7B). Therefore, we infer that lncRNA MIAT may function through dual mechanisms that involve nuclear modulation of miRNA biogenesis and cytoplasmic control of miRNA functional activity, such as through the competing endogenous RNA (ceRNA) network. By integrating bioinformatics predictions with experimental validation, we screened candidate miRNAs spatially interacting and compatible with lncRNA MIAT. The expression of these candidate miRNAs was validated by RT-qPCR (Figures 7C–I). The direct overexpression of MIAT levels *in vivo* led to a reduction in miR-130a-3p within sciatic nerve tissue (Figure 7D). Additionally, EA facilitates repair and consistently downregulates miR-130a-3p (Figure 7E). These findings establish a functional regulatory relationship between MIAT and miR-130a-3p. The miR-130a-3p exhibiting the most significant association with lncRNA MIAT was prioritized for mechanistic investigation. MiR-130a-3p has potential binding sites of lncRNA MIAT (Figure 7P). Dual-luciferase reporter assays demonstrated a direct targeting relationship between lncRNA MIAT and miR-130a-3p (Figure 7Q).

### miR-130a-3p modulates NG108-15 cell viability and neurite growth

Building on previous observations that lncRNA MIAT promotes NG108-15 cell growth and exhibits a targeting relationship with miR-130a-3p, the research team aimed to further investigated whether miR-130a-3p modulates neural cell viability and axonal outgrowth. Conducting comparative analyses of NG108 cells following 24-h treatment with miR-130a-3p mimic, miR-130a-3p inhibitor, and negative control reagent. It was found that overexpression of miR-130a-3p significantly inhibits the cell viability of NG108-15 cells (Figure 7J). Immunofluorescence staining for β3-tubulin further demonstrated that miR-130a-3p mimic transfection significantly suppressed neurite outgrowth in NG108-15 cells (Figures 7K–M). Q-PCR results showed miR-130a-3p mimic significantly downregulated lncRNA MIAT expression in NG108-15 cells (Figure 7N). As demonstrated in Figure 7O, our previous transfection successfully achieved the expected modulation of miR-130a-3p expression levels in NG108-15 cells.

### miR-130a-3p direct interact KLF7

**Figure 8:**
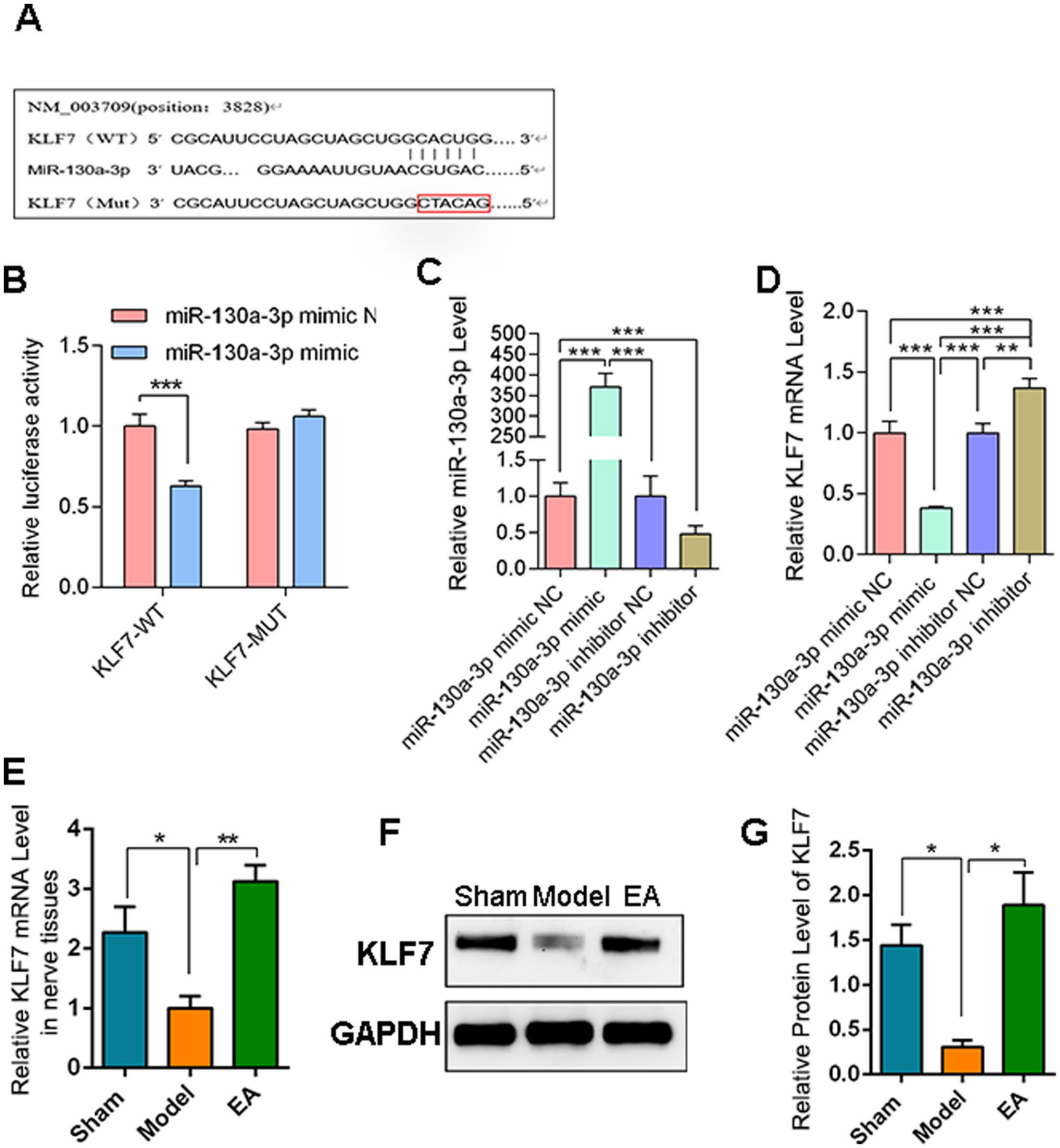
MiR-130a-3p/KLF7C axis participate in EA treatment of PNI. **(A)** Bioinformatic prediction of interaction sites between lncRNA MIAT and KLF7. **(B)** Dual-luciferase reporter assay validating the direct binding of lncRNA MIAT to KLF7 (*n* = 5). **(C)** RT-qPCR analysis of miR-130a-3p expression levels. Data normalized to U6 (*n* = 5). **(D)** RT-qPCR analysis of KLF7 expression on miR-130a-3p transfected NG108-15 cell. Data normalized to GAPDH (*n* = 5). **(E)** RT-qPCR analysis of KLF7 expression levels in PNI tissues. **(F)** Western blot analysis of KLF7 protein expression in PNI tissues. **(G)** Quantitative analysis of KLF7 protein levels (*n* = 5). Data are presented as arithmetic mean ± SEM. ^*^p < 0.05, ^**^p < 0.01, and ^***^p < 0.001.

Bioinformatics prediction analysis revealed putative binding sites between miR-130a-3p and KLF7 (Figure 8A). Co-transfection of the KLF7-WT plasmid and miR-130a-3p mimic resulted in a significant decrease in luciferase activity, confirming the direct interaction between KLF7 and miR-130a-3p (Figure 8B). Following transfection of NG108-15 cells with miR-130a-3p mimic and inhibitor, qPCR analysis revealed significantly downregulated KLF7 mRNA levels in the miR-130a-3p mimic group, whereas KLF7 expression was upregulated in the inhibitor-treated group, demonstrating a significant inverse correlation between miR-130a-3p and KLF7 expression (Figures 8C–D). Finally, *in vivo* validation demonstrated that KLF7 expression was significantly downregulated following PNI, while EA treatment effectively ameliorated this downregulation pattern (Figures 8E–G), which may indicate EA modulates exo-lncRNA MIAT to enhance neuronal cell viability and neurite outgrowth through targeting the miR-130a-3p/KLF7 axis.

## Discussion

Macrophages are innate immune cells which endowed with remarkable plasticity, playing central regulatory roles in host defense mechanisms, tissue repair processes, and homeostasis maintenance (37). After PNI, the number of endoneurial macrophages undergoes a substantial increase and remains elevated for up to 2 weeks after injury (38). Macrophages are observed to initiate phenotypic transition toward M2 polarization at 3 days post-PNI (39). The infiltrating and resident M2 macrophages facilitate axonal regeneration by removing the degenerated axonal and myelin debris (40). Electroacupuncture could regulate macrophage polarization in a wide range of disorders, and it has long been acknowledged in clinical practice as a promising adjunctive treatment modality for promoting neural regeneration and functional recovery (4, 41). However, it remains unclear whether EA promotes peripheral nerve repair through macrophage polarization modulation, and the precise mechanisms driving this process require further investigation. Here, we show that lncRNA MIAT enriched in M2 macrophage-derived exosomes emerge as a critical mediator underlying EA-mediated nerve regeneration of PNI.

In this research, it is find that EA treatment induces significant upregulation of M2 polarization biomarkers in macrophages, while failing to produce significant effects in M1 polarization biomarkers in macrophages, suggesting that EA facilitates peripheral nerve injury repair by modulating macrophage polarization toward to M2 phenotype. It is well established that axonal and myelin regeneration are vital to injured peripheral nerve repair (42). The expansion of M2 macrophage population promotes the secretion of anti-inflammatory factors, which can modulate pro-inflammatory responses, thereby enhancing the intrinsic capacity for axonal regeneration (43).

The polarization of macrophages can be induced by particular group of transcription factors and epigenetic regulation within intercellular communication (44). Exosomes act as vital mediators during intercellular communication by delivering a multifaceted cargo comprising proteins, bioactive lipids, and nucleic acids (including DNA and non-coding RNAs) (45). Emerging evidence indicates that exosomes mediate macrophage polarization via paracrine signaling pathways, which involves the delivery of ncRNAs (46). Studies have shown that endothelial cell-derived exosomes promote macrophage M2 polarization by transmitting lncRNA TUG1 (47). Exosomes derived from angiotensinII-treated cardiomyocytes promote macrophage polarization toward the M1 phenotype by delivering lncRNA PVT1 (48). There are also some researches revealed that exosome lncRNA MALAT1 participates in inducing macrophage M1 polarization (49). Conversely, when human venous endothelial cells are treated with oxidized low-density lipoprotein (ox-LDL), exosomal MALAT1 levels are similarly upregulated, however, it promotes macrophage M2 polarization (50).

Our discovery revealed that exosomal lncRNA MIAT similarly mediates macrophage polarization within injured sciatic nerves. Through systematic validation of lncRNA MIAT expression in serum exosomes and injured peripheral nerve tissues of EA-treated rats, we confirmed the pivotal involvement of MIAT in EA treatment of PNI. Furthermore, when exosome release was inhibited or lncRNA MIAT was knocked down in PNI rats receiving EA intervention, the ameliorative effects of EA on promoting macrophage polarization toward the M2 phenotype were significantly abolished. Our investigation into the regulatory role of lncRNA MIAT in macrophage polarization is consistent with the reports in the literature (51, 52).

This study identifies lncRNA MIAT as a key regulator of M2 polarization, though the precise temporal dynamics of its initial induction by EA require further clarification. The finding that exogenous lncRNA MIAT overexpression is sufficient to trigger M2 polarization suggests it functions as an early-response mediator. It is proposed that EA rapidly activates lncRNA MIAT transcription in local resident macrophages (M0) or recruited monocytes. This initial burst of lncRNA MIAT then derects the downstream polarization program toward the M2 phenotype. Once M2 macrophages are established, they further increase lncRNA MIAT production and secrete it in exosomes, creating a positive feedback loop that stabilizes the M2 state and progressively remodels the injury-site microenvironment to support nerve repair.

Numerous studies have shown that lncRNA MIAT is a promising predictor which is involved in the pathophysiological processes of several diseases, such as cardiovascular diseases, cancer, and neurological disorders (53, 54). Studies confirm that lncRNA MIAT localizes to specific subregions in subpopulations of differentiated murine neurons, and it contributes to neuronal development and survival, while also participates in the regulation of neurogenesis (55). An RNA sequencing studies have revealed a marked downregulation of lncRNA MIAT expression in injured neural tissues (32). lncRNA MIAT has been demonstrated to exert anti-apoptotic effects on neurons. In a study of investigating motor function recovery in spinal cord injury (SCI) rat models, researchers found that overexpressing lncRNA MIAT significantly suppressed neuronal apoptosis (56). Similarly, experimental evidence from another laboratory also substantiated that upregulation of lncRNA MIAT effectively inhibits neuronal apoptosis (57). However, the underlying mechanisms of lncRNA MIAT on neuronal cells remain controversial and require further investigation of specific molecular pathways.

It is generally accepted that the functional mechanisms of lncRNAs are predominantly determined by their subcellular localization, with cytoplasmic localized lncRNAs primarily functioning through the competing endogenous RNA (ceRNA) mechanism (58). The ceRNA mechanism refers to a regulatory process through which lncRNAs competitively bind to microRNA response elements (MREs), consequently sequestering miRNAs and disrupting their post-transcriptional repression of target mRNAs (59). Recently, more and more studies confirmed that lncRNA MIAT can act as miRNA sponges reducing their regulatory effects on the target mRNAs (60, 61). With the analysis utilizing predictive TargetScan, starBase, and miRDB databases, we identified multiple putative binding sites between lncRNA MIAT and miR-130a-3p. This bioinformatic prediction was subsequently validated through dual-luciferase reporter gene assays. Notably, prior studies have documented that lncRNA MIAT functions as a ceRNA for miR-130a-3p, regulating diabetic nephropathy podocyte injury (62).

This study demonstrated the functional interplay between lncRNA MIAT and miR-130a-3p. Through *in vitro* experiments involving transfection of NG108-15 neuronal cells with miR-130a-3p mimic and inhibitor, and the study further revealed that lncRNA MIAT exerts regulatory effects on PNI repair by targeting miR-130a-3p. These results will contribute to elucidating the complete ceRNA network of lncRNA MIAT.

KLF7 is a key transcription factor in axon outgrowth (63). The depletion of KLF7 impedes axon growth (64). Research showed that KLF7 could regulate neuroapoptosis and inflammation response (65). Overexpression of KLF7 ameliorated motor function in traumatic brain injury (TBI) mice, and alleviating the myelin rarefaction (66). In this study, miR-130a-3p is identified as a potential regulator targeting KLF7 through bioinformatics analysis, and proved that miR-130a-3p modulate KLF7 expression to influence neuronal growth. Independent evidence underscores the importance of miR-130a-3p and KLF7 in macrophage polarization. While in ischemic stroke KLF7 drives M2 polarization (67); in sepsis-associated encephalopathy, direct inhibition of miR-130a-3p is the key to promoting the M2 phenotype (68). Both findings point to a common regulatory nexus. Therefore, it is plausible that lncRNA MIAT in macrophages exploits the miR-130a-3p/KLF7 axis to reinforce the polarization of M2.

The causal role of the lncRNA MIAT/miR-130a-3p/KLF7 axis is supported by various pieces of evidence. Initially, manipulating lncRNA MIAT *in vivo* directly influenced the expression of miR-130a-3p. Subsequently, molecular interactions were verified at every stage of the axis. Moreover, EA consistently triggered an expression cascade in damaged nerve tissue, marked by elevated lncRNA MIAT, reduced miR-130a-3p, and heightened KLF7 levels.

Current interventions for nerve injuries, including surgical, pharmacological, and rehabilitative approaches, focus on restoring function or relieving symptoms rather than promoting structural nerve regeneration (69, 70). As a result, therapeutic outcomes are often suboptimal and transient (71). Our research identifies a unique pathway that directly influences axonal regrowth and remyelination. This discovery positions the lncRNA MIAT/miR-130a-3p/KLF7 axis not just as a symptomatic treatment option but as a potential regenerative strategy.

Laying the groundwork for clinical translation represents a fundamental aim of basic scientific inquiry, a principle that equally applies to mechanistic studies of acupuncture. Yin et al. (72) identified the novel druggable target transgelin-2 based on the clinical efficacy of acupuncture in treating asthma. This discovery exemplifies a translational journey that moves from acupuncture mechanistic research to drug discovery. Similarly, our study similarly opens a translational pathway, suggesting that molecules in the lncRNA MIAT/miR-130a-3p/KLF7 axis may be therapeutic targets for future clinical use. This potential is reinforced by advanced molecular delivery systems (73), enabling precise lncRNA MIAT overexpression to enhance nerve regeneration and repair.

This study uncovers a promising mechanism of EA. However, a critical evaluation of its broader clinical application for PNI is necessary. Although EA offers significant advantages as a potential adjunctive or complementary therapy, including its non-pharmacological nature and minimal side effects, its efficacy may be affected by practitioner skill, the absence of standardized parameters, and patient variability. Consequently, research into the mechanisms of acupuncture can address these issues by providing a molecular foundation that reduces reliance on empiricism and guides the optimization of standardized, reproducible EA protocols.

### Limitation

Although the present research has elucidated the mechanism by which EA regulates M2-exo-lncRNA MIAT to enhance neuronal cell viability and neurite outgrowth, certain methodological limitations must be acknowledged. A key limitation is that the experimental design cannot determine whether electroacupuncture directly upregulates lncRNA MIAT within macrophages or whether macrophages acquire it from other cells *in vivo*. Therefore, the precise cellular source of lncRNA MIAT requires further investigation. In future work, appropriate tools should be employed to further clarify the origin of lncRNA MIAT. Secondly, the MIAT/miR-130a-3p/KLF7 axis may constitute one of several pathways within the extensive regulatory network of EA. Due to the significant crosstalk among signaling pathways and EA’s capacity for multi-pathway modulation, future studies should investigate the comprehensive regulatory network of EA to elucidate the systemic mechanisms underlying EA-driven PNI repair.

## Conclusion

In conclusion, this study reveals a novel mechanism for EA in peripheral nerve repair. We found that EA acts through M2 macrophage-derived exosomes, which deliver lncRNA MIAT to promote neurite outgrowth via the miR-130a-3p/KLF7 axis. These findings suggest that EA does not merely modulate nerve activity. Instead, it actively initiates a cascade where immune cells release exosomes to promote regeneration. and identify the MIAT/miR-130a-3p/KLF7 axis as a novel therapeutic target. Future work requires validation in independent models, investigation in chronic neuropathic conditions, and the development of targeted delivery strategies to advance this mechanistic insight toward therapy.

## Data Availability

The original contributions presented in the study are included in the article/supplementary material, further inquiries can be directed to the corresponding authors.

## References

[1] Aonuma T Moukette B Kawaguchi S Barupala NP Sepúlveda MN Frick K . MiR-150 attenuates maladaptive cardiac remodeling mediated by long noncoding RNA MIAT and directly represses profibrotic Hoxa4. Circ Heart Fail. (2022) 15:e008686. doi: 10.1161/CIRCHEARTFAILURE.121.008686, 35000421 PMC9018469

[2] Idrisova KF Zeinalova AK Masgutova GA Bogov AA Allegrucci C Syromiatnikova VY . Application of neurotrophic and proangiogenic factors as therapy after peripheral nervous system injury. Neural Regen Res. (2022) 17:1240–7. doi: 10.4103/1673-5374.327329, 34782557 PMC8643040

[3] Ruijs AC Jaquet JB Kalmijn S Giele H Hovius SE. Median and ulnar nerve injuries: a meta-analysis of predictors of motor and sensory recovery after modern microsurgical nerve repair. Plast Reconstr Surg. (2005) 116:484–94. doi: 10.1097/01.prs.0000172896.86594.07, 16079678

[4] Friedemann T Kark E Cao N Klaßen M Meyer-Hamme G Greten JH . Acupuncture improves chemotherapy-induced neuropathy explored by neurophysiological and clinical outcomes - the randomized, controlled, cross-over ACUCIN trial. Phytomedicine. (2022) 104:154294. doi: 10.1016/j.phymed.2022.154294, 35785559

[5] Yang Y Rao C Yin T Wang S Shi H Yan X . Application and underlying mechanism of acupuncture for the nerve repair after peripheral nerve injury: remodeling of nerve system. Front Cell Neurosci. (2023) 17:1253438. doi: 10.3389/fncel.2023.1253438, 37941605 PMC10627933

[6] Hu LN Tian JX Gao W Zhu J Mou FF Ye XC . Electroacupuncture and moxibustion promote regeneration of injured sciatic nerve through Schwann cell proliferation and nerve growth factor secretion. Neural Regen Res. (2018) 13:477–83. doi: 10.4103/1673-5374.228731, 29623933 PMC5900511

[7] Liu YP Luo ZR Wang C Cai H Zhao TT Li H . Electroacupuncture promoted nerve repair after peripheral nerve injury by regulating miR-1b and its target brain-derived neurotrophic factor. Front Neurosci. (2020) 14:525144. doi: 10.3389/fnins.2020.525144, 33132818 PMC7550428

[8] Tian MY Yang YD Qin WT Liu BN Mou FF Zhu J . Electroacupuncture promotes nerve regeneration and functional recovery through regulating lncRNA GAS5 targeting miR-21 after sciatic nerve injury. Mol Neurobiol. (2024) 61:935–49. doi: 10.1007/s12035-023-03613-3, 37672149 PMC10861712

[9] Wang J Liu JJ Tang ZY Liang QQ Cui JW. Acupuncture promotes neurological recovery and regulates lymphatic function after acute inflammatory nerve root injury. Heliyon. (2024) 10:e35702. doi: 10.1016/j.heliyon.2024.e35702, 39229545 PMC11369430

[10] Zhao S Wang S Cao L Zeng H Lin S Lin Z . Acupuncture promotes nerve repair through the benign regulation of mTOR-mediated neuronal autophagy in traumatic brain injury rats. CNS Neurosci Ther. (2023) 29:458–70. doi: 10.1111/cns.14018, 36422883 PMC9804054

[11] Du F Liu S. Electroacupuncture with high frequency at acupoint ST-36 induces regeneration of lost enteric neurons in diabetic rats via GDNF and PI3K/AKT signal pathway. Am J Physiol Regul Integr Comp Physiol. (2015) 309:R109–18. doi: 10.1152/ajpregu.00396.2014, 25972459

[12] Elberg G Liraz-Zaltsman S Reichert F Matozaki T Tal M Rotshenker S. Deletion of SIRPα (signal regulatory protein-α) promotes phagocytic clearance of myelin debris in Wallerian degeneration, axon regeneration, and recovery from nerve injury. J Neuroinflammation. (2019) 16:277. doi: 10.1186/s12974-019-1679-x, 31883525 PMC6935070

[13] Zigmond RE Echevarria FD. Macrophage biology in the peripheral nervous system after injury. Prog Neurobiol. (2019) 173:102–21. doi: 10.1016/j.pneurobio.2018.12.001, 30579784 PMC6340791

[14] Tomlinson JE Žygelytė E Grenier JK Edwards MG Cheetham J. Temporal changes in macrophage phenotype after peripheral nerve injury. J Neuroinflammation. (2018) 15:185. doi: 10.1186/s12974-018-1219-0, 29907154 PMC6003127

[15] Ren X Gao X Li Z Ding Y Xu A Du L . Electroacupuncture ameliorates neuroinflammation by inhibiting TRPV4 channel in ischemic stroke. CNS Neurosci Ther. (2024) 30:e14618. doi: 10.1111/cns.14618, 38334061 PMC10853892

[16] Wang HF Chen L Xie Y Wang XF Yang K Ning Y . Electroacupuncture facilitates M2 macrophage polarization and its potential role in the regulation of inflammatory response. Biomed Pharmacother. (2021) 140:111655. doi: 10.1016/j.biopha.2021.111655, 34029955

[17] Xu X Huang X Xiao L Wang J Yang X Wu Y. Mechanism of electro-acupuncture in alleviating intestinal injury in septic mice via polyamine-related M2-macrophage polarization. Front Immunol. (2024) 15:1373876. doi: 10.3389/fimmu.2024.1373876, 38715602 PMC11075497

[18] Kalluri R LeBleu VS. The biology, function, and biomedical applications of exosomes. Science. (2020) 367:eaau6977. doi: 10.1126/science.aau6977, 32029601 PMC7717626

[19] Yáñez-Mó M Siljander PR Andreu Z Zavec AB Borràs FE Buzas EI . Biological properties of extracellular vesicles and their physiological functions. J Extracell Vesicles. (2015) 4:27066. doi: 10.3402/jev.v4.27066, 25979354 PMC4433489

[20] Dai Y Wang S Chang S Ren D Shali S Li C . M2 macrophage-derived exosomes carry microRNA-148a to alleviate myocardial ischemia/reperfusion injury via inhibiting TXNIP and the TLR4/NF-κB/NLRP3 inflammasome signaling pathway. J Mol Cell Cardiol. (2020) 142:65–79. doi: 10.1016/j.yjmcc.2020.02.007, 32087217

[21] Momen-Heravi F Bala S Bukong T Szabo G. Exosome-mediated delivery of functionally active miRNA-155 inhibitor to macrophages. Nanomedicine. (2014) 10:1517–27. doi: 10.1016/j.nano.2014.03.014, 24685946 PMC4180003

[22] Lyu Q Shi LQ Chen HY Lu M Liang XC Ma XD . Electroacupuncture combined with NSCs-Exo alters the response of hippocampal neurons in a chronic unpredictable mild stress paradigm in ovx rats. Life Sci. (2024) 359:123235. doi: 10.1016/j.lfs.2024.123235, 39528081

[23] Liu Y Li YP Xiao LM Chen LK Zheng SY Zeng EM . Extracellular vesicles derived from M2 microglia reduce ischemic brain injury through microRNA-135a-5p/TXNIP/NLRP3 axis. Lab Investig. (2021) 101:837–50. doi: 10.1038/s41374-021-00545-1, 33875790

[24] Luo Z Peng W Xu Y Xie Y Liu Y Lu H . Exosomal OTULIN from M2 macrophages promotes the recovery of spinal cord injuries via stimulating Wnt/β-catenin pathway-mediated vascular regeneration. Acta Biomater. (2021) 136:519–32. doi: 10.1016/j.actbio.2021.09.026, 34551329

[25] Li H Feng Y Zheng X Jia M Mei Z Wang Y . M2-type exosomes nanoparticles for rheumatoid arthritis therapy via macrophage re-polarization. J Control Release. (2022) 341:16–30. doi: 10.1016/j.jconrel.2021.11.019, 34793917

[26] Wang D Liu Y Diao S Shan L Zhou J. Long non-coding RNAs within macrophage-derived exosomes promote BMSC osteogenesis in a bone fracture rat model. Int J Nanomedicine. (2023) 18:1063–83. doi: 10.2147/IJN.S398446, 36879890 PMC9985426

[27] Wang Y Lin Q Zhang H Wang S Cui J Hu Y . M2 macrophage-derived exosomes promote diabetic fracture healing by acting as an immunomodulator. Bioact Mater. (2023) 28:273–83. doi: 10.1016/j.bioactmat.2023.05.018, 37303851 PMC10247878

[28] Chen J Zhou R Liang Y Fu X Wang D Wang C. Blockade of lncRNA-ASLNCS5088-enriched exosome generation in M2 macrophages by GW4869 dampens the effect of M2 macrophages on orchestrating fibroblast activation. FASEB J. (2019) 33:12200–12. doi: 10.1096/fj.201901610, 31373848 PMC6902732

[29] Zhao X Deng H Feng Y Wang Y Yao X Ma Y . Immune-cell-mediated tissue engineering strategies for peripheral nerve injury and regeneration. J Mater Chem B. (2024) 12:2217–35. doi: 10.1039/d3tb02557h, 38345580

[30] Guo C Li J Guo M Bai R Lei G Sun H . Aberrant expressions of MIAT and PVT1 in serum exosomes of schizophrenia patients. Schizophr Res. (2022) 240:71–2. doi: 10.1016/j.schres.2021.12.013, 34959074

[31] Babu S Krishnan M Panneerselvam A Chinnaiyan M. A comprehensive review on therapeutic application of mesenchymal stem cells in neuroregeneration. Life Sci. (2023) 327:121785. doi: 10.1016/j.lfs.2023.121785, 37196856

[32] Arthur-Farraj PJ Morgan CC Adamowicz M Gomez-Sanchez JA Fazal SV Beucher A . Changes in the coding and non-coding transcriptome and DNA methylome that define the Schwann cell repair phenotype after nerve injury. Cell Rep. (2017) 20:2719–34. doi: 10.1016/j.celrep.2017.08.064, 28903050 PMC5608958

[33] Peng L Chen Y Ou Q Wang X Tang N. lncRNA MIAT correlates with immune infiltrates and drug reactions in hepatocellular carcinoma. Int Immunopharmacol. (2020) 89:107071. doi: 10.1016/j.intimp.2020.107071, 33221703

[34] Li JH Liu S Zhou H Qu LH Yang JH. starBase v2.0: decoding miRNA-ceRNA, miRNA-ncRNA and protein-RNA interaction networks from large-scale CLIP-Seq data. Nucleic Acids Res. (2014) 42:D92–7. doi: 10.1093/nar/gkt1248, 24297251 PMC3964941

[35] China Association of Acupuncture and Moxibustion. Nomenclature and location of acupuncture points for laboratory animals part 2: rat. World J Acupunct Moxibustion. (2025) 35:163–5. doi: 10.1016/j.wjam.2024.12.003

[36] Giacca G Naldini L Squadrito ML. Harnessing lentiviral vectors for *in vivo* gene therapy of liver metastases. Clin Transl Med. (2024) 14:e1542. doi: 10.1002/ctm2.1542, 38230542 PMC10792462

[37] Park MD Silvin A Ginhoux F Merad M. Macrophages in health and disease. Cell. (2022) 185:4259–79. doi: 10.1016/j.cell.2022.10.007, 36368305 PMC9908006

[38] Hellenbrand DJ Quinn CM Piper ZJ Morehouse CN Fixel JA Hanna AS. Inflammation after spinal cord injury: a review of the critical timeline of signaling cues and cellular infiltration. J Neuroinflammation. (2021) 18:284. doi: 10.1186/s12974-021-02337-2, 34876174 PMC8653609

[39] Gordon S Martinez FO. Alternative activation of macrophages: mechanism and functions. Immunity. (2010) 32:593–604. doi: 10.1016/j.immuni.2010.05.007, 20510870

[40] Stratton JA Holmes A Rosin NL Sinha S Vohra M Burma NE . Macrophages regulate Schwann cell maturation after nerve injury. Cell Rep. (2018) 24:2561–2572.e6. doi: 10.1016/j.celrep.2018.08.004, 30184491

[41] Huang MC Chang SC Liao WL Ke TW Lee AL Wang HM . Acupuncture may help to prevent chemotherapy-induced peripheral neuropathy: a randomized, sham-controlled, single-blind study. Oncologist. (2023) 28:e436–47. doi: 10.1093/oncolo/oyad065, 36971468 PMC10243779

[42] Li C Liu SY Zhou LP Min TT Zhang M Pi W . Polydopamine-modified chitin conduits with sustained release of bioactive peptides enhance peripheral nerve regeneration in rats. Neural Regen Res. (2022) 17:2544–50. doi: 10.4103/1673-5374.339006, 35535909 PMC9120711

[43] Kigerl KA Gensel JC Ankeny DP Alexander JK Donnelly DJ Popovich PG. Identification of two distinct macrophage subsets with divergent effects causing either neurotoxicity or regeneration in the injured mouse spinal cord. J Neurosci. (2009) 29:13435–44. doi: 10.1523/jneurosci.3257-09.2009, 19864556 PMC2788152

[44] Locati M Curtale G Mantovani A. Diversity, mechanisms, and significance of macrophage plasticity. Annu Rev Pathol. (2020) 15:123–47. doi: 10.1146/annurev-pathmechdis-012418-012718, 31530089 PMC7176483

[45] Gowda R Robertson BM Iyer S Barry J Dinavahi SS Robertson GP. The role of exosomes in metastasis and progression of melanoma. Cancer Treat Rev. (2020) 85:101975. doi: 10.1016/j.ctrv.2020.101975, 32050108

[46] Ahmad I Valverde A Naqvi RA Naqvi AR. Long non-coding RNAs RN7SK and GAS5 regulate macrophage polarization and innate immune responses. Front Immunol. (2020) 11:604981. doi: 10.3389/fimmu.2020.604981, 33362791 PMC7757381

[47] Ma W Zhang W Cui B Gao J Liu Q Yao M . Functional delivery of lncRNA TUG1 by endothelial progenitor cells derived extracellular vesicles confers anti-inflammatory macrophage polarization in sepsis via impairing miR-9-5p-targeted SIRT1 inhibition. Cell Death Dis. (2021) 12:1056. doi: 10.1038/s41419-021-04117-5, 34743197 PMC8572288

[48] Cao F Li Z Ding W Yan L Zhao Q. Angiotensin II-treated cardiac myocytes regulate M1 macrophage polarization via transferring exosomal PVT1. J Immunol Res. (2021) 2021:1994328. doi: 10.1155/2021/1994328, 34514000 PMC8427676

[49] Liu J Niu Z Zhang R Peng Z Wang L Liu Z . MALAT1 shuttled by extracellular vesicles promotes M1 polarization of macrophages to induce acute pancreatitis via miR-181a-5p/HMGB1 axis. J Cell Mol Med. (2021) 25:9241–54. doi: 10.1111/jcmm.16844, 34448533 PMC8500974

[50] Huang C Han J Wu Y Li S Wang Q Lin W . Exosomal MALAT1 derived from oxidized low-density lipoprotein-treated endothelial cells promotes M2 macrophage polarization. Mol Med Rep. (2018) 18:509–15. doi: 10.3892/mmr.2018.8982, 29750307

[51] Li J Zou CL Zhang ZM Xue F. Knockdown of lncRNA MIAT attenuated lipopolysaccharide-induced microglial cells injury by sponging miR-613. Mamm Genome. (2022) 33:471–9. doi: 10.1007/s00335-022-09946-z, 35079871

[52] Wang Z Kun Y Lei Z Dawei W Lin P Jibo W. lncRNA MIAT downregulates IL-1β, TNF-ɑ to suppress macrophage inflammation but is suppressed by ATP-induced NLRP3 inflammasome activation. Cell Cycle. (2021) 20:194–203. doi: 10.1080/15384101.2020.1867788, 33459112 PMC7889136

[53] Da CM Gong CY Nan W Zhou KS Wu ZL Zhang HH. The role of long non-coding RNA MIAT in cancers. Biomed Pharmacother. (2020) 129:110359. doi: 10.1016/j.biopha.2020.110359, 32535389

[54] Sun C Huang L Li Z Leng K Xu Y Jiang X . Long non-coding RNA MIAT in development and disease: a new player in an old game. J Biomed Sci. (2018) 25:23. doi: 10.1186/s12929-018-0427-3, 29534728 PMC5851271

[55] Aprea J Prenninger S Dori M Ghosh T Monasor LS Wessendorf E . Transcriptome sequencing during mouse brain development identifies long non-coding RNAs functionally involved in neurogenic commitment. EMBO J. (2013) 32:3145–60. doi: 10.1038/emboj.2013.245, 24240175 PMC3981144

[56] Li D Yang T Shao C Cao Z Zhang H. lncRNA MIAT activates vascular endothelial growth factor A through RAD21 to promote nerve injury repair in acute spinal cord injury. Mol Cell Endocrinol. (2021) 528:111244. doi: 10.1016/j.mce.2021.111244, 33741460

[57] Mirzaei H Salehi A Javan B Enayati A Nabi MO Zahedi M . *Potentilla reptans* L. preconditioning regulates H19 and MIAT long noncoding RNAs in H9C2 myoblasts ischemia/reperfusion model. BMC Complement Med Ther. (2023) 23:272. doi: 10.1186/s12906-023-04071-z, 37525174 PMC10388489

[58] Tay Y Rinn J Pandolfi PP. The multilayered complexity of ceRNA crosstalk and competition. Nature. (2014) 505:344–52. doi: 10.1038/nature12986, 24429633 PMC4113481

[59] Bossi L Figueroa-Bossi N. Competing endogenous RNAs: a target-centric view of small RNA regulation in bacteria. Nat Rev Microbiol. (2016) 14:775–84. doi: 10.1038/nrmicro.2016.129, 27640758

[60] Chen Y Chen X Li H Li Y Cheng D Tang Y . Serum extracellular vesicles containing MIAT induces atrial fibrosis, inflammation and oxidative stress to promote atrial remodeling and atrial fibrillation via blockade of miR-485-5p-mediated CXCL10 inhibition. Clin Transl Med. (2021) 11:e482. doi: 10.1002/ctm2.482, 34459123 PMC8329545

[61] Yan B Yao J Liu JY Li XM Wang XQ Li YJ . lncRNA-MIAT regulates microvascular dysfunction by functioning as a competing endogenous RNA. Circ Res. (2015) 116:1143–56. doi: 10.1161/CIRCRESAHA.116.305510, 25587098

[62] Zhang M Zhao S Xu C Shen Y Huang J Shen S . Ablation of lncRNA MIAT mitigates high glucose-stimulated inflammation and apoptosis of podocyte via miR-130a-3p/TLR4 signaling axis. Biochem Biophys Res Commun. (2020) 533:429–36. doi: 10.1016/j.bbrc.2020.09.034, 32972755

[63] Lei L Laub F Lush M Romero M Zhou J Luikart B . The zinc finger transcription factor Klf7 is required for TrkA gene expression and development of nociceptive sensory neurons. Genes Dev. (2005) 19:1354–64. doi: 10.1101/gad.1227705, 15937222 PMC1142558

[64] Smaldone S Ramirez F. Multiple pathways regulate intracellular shuttling of MoKA, a co-activator of transcription factor KLF7. Nucleic Acids Res. (2006) 34:5060–8. doi: 10.1093/nar/gkl659, 16990251 PMC1636432

[65] Feng W Chen J Huang W Wang G Chen X Duan L . HMGB1-mediated elevation of KLF7 facilitates hepatocellular carcinoma progression and metastasis through upregulating TLR4 and PTK2. Theranostics. (2023) 13:4042–58. doi: 10.7150/thno.84388, 37554278 PMC10405848

[66] Li WY Fu XM Wang ZD Li ZG Ma D Sun P . Krüppel-like factor 7 attenuates hippocampal neuronal injury after traumatic brain injury. Neural Regen Res. (2022) 17:661–72. doi: 10.4103/1673-5374.320991, 34380908 PMC8504401

[67] Wei R Li S Tang L. KLF7 blocks MKNK2/HIF-1 pathway-mediated M1 microglia polarization to ameliorate ischemic stroke-induced neurological injury. Brain Behav. (2025) 15:e70850. doi: 10.1002/brb3.70850, 40923147 PMC12417964

[68] Peng LS Xu Y Wang QS. YY1 promotes microglia M2 polarization through the MIR-130A-3P/TREM-2 AXIS to alleviate SEPSIS-associated encephalopathy. Shock. (2022) 58:128–36. doi: 10.1097/shk.0000000000001914, 35234205

[69] Chiaramonte R Romano M Vecchio M. A systematic review of the diagnostic methods of small fiber neuropathies in rehabilitation. Diagnostics. (2020) 10:613. doi: 10.3390/diagnostics10090613, 32825514 PMC7554909

[70] Kong Y Pan T Kuss M Yang K John JV Duan B. Enhancing peripheral nerve regeneration with rehabilitation and biomaterial-driven drug delivery strategies. Prog Biomed Eng. (2025) 7:042003. doi: 10.1088/2516-1091/adf7ee PMC13063242 40763793

[71] Vecchio M Chiaramonte R Romano M Pavone P Musumeci G Mauro GL. A systematic review of pharmacologic and rehabilitative treatment of small fiber neuropathies. Diagnostics. (2020) 10:1022. doi: 10.3390/diagnostics10121022, 33260566 PMC7761307

[72] Yin LM Xu YD Peng LL Duan TT Liu JY Xu Z . Transgelin-2 as a therapeutic target for asthmatic pulmonary resistance. Sci Transl Med. (2018) 10:eaam8604. doi: 10.1126/scitranslmed.aam8604, 29437149 PMC6310021

[73] Kim JE Ji YE Hwang HJ Go GE Lim HJ Yoo J . Engineered MSC-EVs loaded with BDNF-enhancing neuropeptides via a non-disruptive method enhance post-stroke neuroregeneration via intranasal delivery. J Nanobiotechnology. (2025) 23:594. doi: 10.1186/s12951-025-03654-x, 40883738 PMC12395722

